# Graphene/MoS_2_/FeCoNi(OH)_x_ and Graphene/MoS_2_/FeCoNiP_x_ multilayer-stacked vertical nanosheets on carbon fibers for highly efficient overall water splitting

**DOI:** 10.1038/s41467-021-21742-y

**Published:** 2021-03-02

**Authors:** Xixi Ji, Yanhong Lin, Jie Zeng, Zhonghua Ren, Zijia Lin, Yongbiao Mu, Yejun Qiu, Jie Yu

**Affiliations:** Shenzhen Engineering Lab for Supercapacitor Materials, Shenzhen Key Laboratory for Advanced Materials, School of Material Science and Engineering, Harbin Institute of Technology, Shenzhen, University Town, Shenzhen, China

**Keywords:** Materials chemistry, Materials for energy and catalysis, Nanoscale materials

## Abstract

Development of excellent and cheap electrocatalysts for water electrolysis is of great significance for application of hydrogen energy. Here, we show a highly efficient and stable oxygen evolution reaction (OER) catalyst with multilayer-stacked hybrid structure, in which vertical graphene nanosheets (VGSs), MoS_2_ nanosheets, and layered FeCoNi hydroxides (FeCoNi(OH)_x_) are successively grown on carbon fibers (CF/VGSs/MoS_2_/FeCoNi(OH)_x_). The catalyst exhibits excellent OER performance with a low overpotential of 225 and 241 mV to attain 500 and 1000 mA cm^−2^ and small Tafel slope of 29.2 mV dec^−1^. Theoretical calculation indicates that compositing of FeCoNi(OH)_x_ with MoS_2_ could generate favorable electronic structure and decrease the OER overpotential, promoting the electrocatalytic activity. An alkaline water electrolyzer is established using CF/VGSs/MoS_2_/FeCoNi(OH)_x_ anode for overall water splitting, which generates a current density of 100 mA cm^−2^ at 1.59 V with excellent stability over 100 h. Our highly efficient catalysts have great prospect for water electrolysis.

## Abstract (web summary)

While water-splitting electrocatalysis offers a renewable means for carbon-neutral energy production, it is a challenge to design efficient, active, and stable catalysts. Here, authors prepare multilayer composite nanosheet materials as bifunctional water-splitting electrocatalysts.

## Introduction

With increasing concerns about energy crises and environmental pollution, intensive efforts have been made to develop sustainable and clean energy^1^. Among the different renewable energy sources, hydrogen is more appreciated due to its zero carbon emission and high energy density. The hydrogen energy has been widely studied and used since water splitting was first reported in 1789^2^. The water splitting process includes hydrogen evolution reaction (HER) and oxygen evolution reaction (OER), among which the OER is much harsher than HER because of its sluggish four electron process^3^. Conventionally, Pt-, Ir-, and Ru-based electrocatalysts are used for water splitting due to their good HER and OER performances. However, widespread application is difficult for these noble metal catalysts because of their scarcity and high cost. Therefore, it is of great importance to develop electrocatalysts for water splitting with low cost, abundance in raw materials, and large output.

Recently, much attention has been paid to the study of non-noble metal electrocatalysts such as hydroxides, sulfides, and phosphides of non-noble metals^4^. Two-dimensional layered structure is favorable for applications of energy storage and catalysts due to the advantages of in-plain electron transfer mode, abundant active sites at edges, and high specific surface area^5–9^. Layered metal hydroxides are considered to be promising materials for OER due to the demonstrated excellent performance^10–14^. For example, composite nanotubes containing two phases of (NiCo)_0.85_Se and NiCo layered double hydroxides (LDH) grown on carbon cloth show an overpotential of 216 mV at a current density of 10 mA cm^−2^ as catalysts for OER^11^. For LDH-based catalysts NiFe^−^OH/NiFeP/NF^12^ and Co(OH)_2_@NCNT@NF^13^, the current density of 100 mA cm^−2^ has been achieved for OER at 1.46 and 1.64 V, respectively. However, further improvement of the catalysts for OER is still necessary to satisfy the requirements of large current density (>500 mA cm^−2^) at low overpotential (<300 mV), low cost, and high yield production^15^. With respect to the HER catalysts, recent literatures indicate that MoS_2_ and metal phosphides are more promising^16–20^. However, their performances are still not good enough when applied for overall water splitting in alkaline electrolyte, where the voltage achieving 100 mA cm^−2^ is still too high to meet the requirements of practical applications^3,21^. Although many catalysts may have high catalytic activity themselves there still exists different disadvantages during application such as poor conductivity, limited reaction sites, and slow reaction kinetics. It has been found that in addition to compensating shortcomings each other the combination of different catalysts or materials may cause synergistic effects and generates much enhanced performance than the single components^10^. Therefore, a rational design of composite or hybrid structure is important for achieving high catalytic performance by ensuring full exposure towards the electrolyte and easy gas release, providing rapid and efficient charge transfer paths, and possible synergistic effects. To this end, vertical graphene sheets (VGSs) may be a good choice as the substrates of the active materials such as metal hydroxides and MoS_2_ due to their high electrical conductivity, abundant edges, and well-aligned and dispersed structure.

Based on the above consideration, in this work, we show a composite structure with the graphene sheets, MoS_2_ nanosheets, and FeCoNi(OH)_x_ or FeCoNiP_x_ nanosheets successively stack on carbon fibers (CFs) vertically, which are prepared via thermal chemical vapor deposition (CVD) and electrodeposition. The CF/VGSs/MoS_2_/FeCoNi(OH)_x_ (CF/VMFO) composite electrode shows an excellent OER performance in 1 M KOH solution, achieving a current density of 500 mA cm^−2^ at the overpotential of 225 mV and a small Tafel slope of 29.2 mV dec^−1^, which is better than that of the most reported catalysts. The CF/VGSs/MoS_2_/FeCoNiP_x_ (CF/VMFP) composite electrode is obtained by phosphating the FeCoNi(OH)_x_ nanosheets, which exhibits good HER performance in 1 M KOH solution with an overpotential of 43 mV at 10 mA cm^−2^ and Tafel slope of 25.2 mV dec^−1^. When using the CF/VMFO and CF/VMFP as anode and cathode for overall water splitting, a high current density of 100 mA cm^−2^ is delivered at 1.59 V.

## Results

### Preparation and characterization of materials

**Fig. 1:**
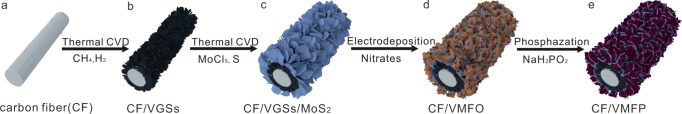
A schematic diagram showing preparation process of samples. **a**–**d** Preparation procedure of CF/VMFO. **e** Preparation procedure of CF/VMFP from CF/VMFO.

The preparation process of the CF/VMFO includes growth of the VGSs on CFs by thermal CVD, growth of the MoS_2_ nanosheets on the VGSs by thermal CVD, and growth of the FeCoNi(OH)_x_ nanosheets on the MoS_2_ nanosheets by electrodeposition (see Fig. 1 and Experimental section). As demonstrated in our previous paper^22^, the CF/VGSs can be produced in large scale easily by thermal CVD. The FeCoNiP_x_ nanosheets were obtained by phosphating the FeCoNi(OH)_x_ nanosheets. It was observed that with formation of the VGSs, MoS_2_, FeCoNi(OH)_x_, and FeCoNiP_x_ nanosheets the samples became dark black, dark blue, brown, and black successively (Supplementary Fig. 1).

**Fig. 2:**
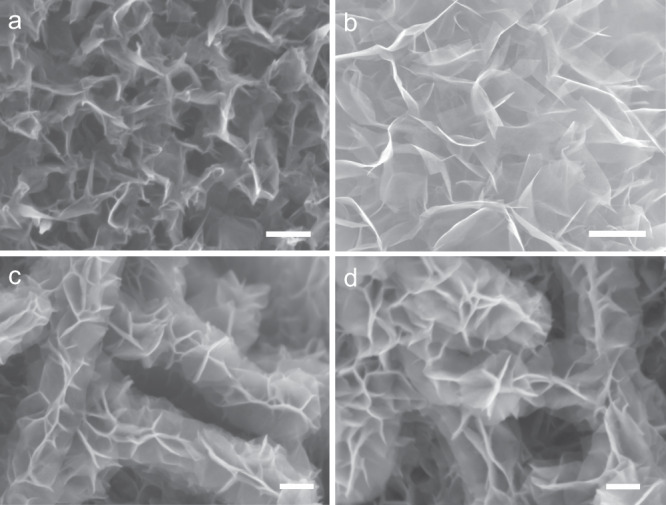
SEM images of different samples. **a** CF/VGSs. **b** CF/VGSs/MoS_2_. **c** CF/VMFO. **d** CF/VMFP. Scale bars: **a** 200 nm; **b** 1 μm; **c**, **d** 200 nm.

The morphology of the different samples was observed by scanning electron microscopy (SEM) (Fig. 2 and Supplementary Fig. 2). The average diameter of the original CFs is about 10.8 μm, which turned into 11.8, 12.3, and 12.7 μm after growing the VGSs, MoS_2_ nanosheets, and FeCoNiP_x_ nanosheets (Supplementary Fig. 2). The VGSs grown on the CFs are vertical and uniform with the edges fully exposed on the surface, which interconnect to form porous structure with the pore size in the range of 50–120 nm (Fig. 2a). As shown in Fig. 2b, after growing the MoS_2_ nanosheets, the morphology of the VGSs disappeared and all the surface is covered by the MoS_2_ nanosheets. The size of MoS_2_ nanosheets is 1–2 μm and the pore size formed by the interconnected MoS_2_ nanosheets is 200–950 nm. The SEM image of the CF/VMFO is shown in Fig. 2c, which shows different morphology from the MoS_2_ nanosheets. It is observed that the FeCoNi(OH)_x_ nanosheets are grown both on the side and top surface of the MoS_2_ nanosheets, which grow both outward and upward and present the outline of MoS_2_ nanosheets (see also Supplementary Fig. 5). The FeCoNi(OH)_x_ nanosheets are denser than the MoS_2_ nanosheets. Obviously, much higher area density of the FeCoNi(OH)_x_ nanosheets can be achieved by growing on MoS_2_ nanosheets than on flat substrates. From the cross-sectional SEM images, the average heights of the VGSs, VGSs/MoS_2_, and VGSs/MoS_2_/FeCoNi(OH)_x_ nanosheet layers are about 530, 620, and 660 nm (Supplementary Fig. 3). As shown in Fig. 2d, the FeCoNiP_x_ nanosheets maintain the morphology of the FeCoNi(OH)_x_ nanosheets after phosphating.

**Fig. 3:**
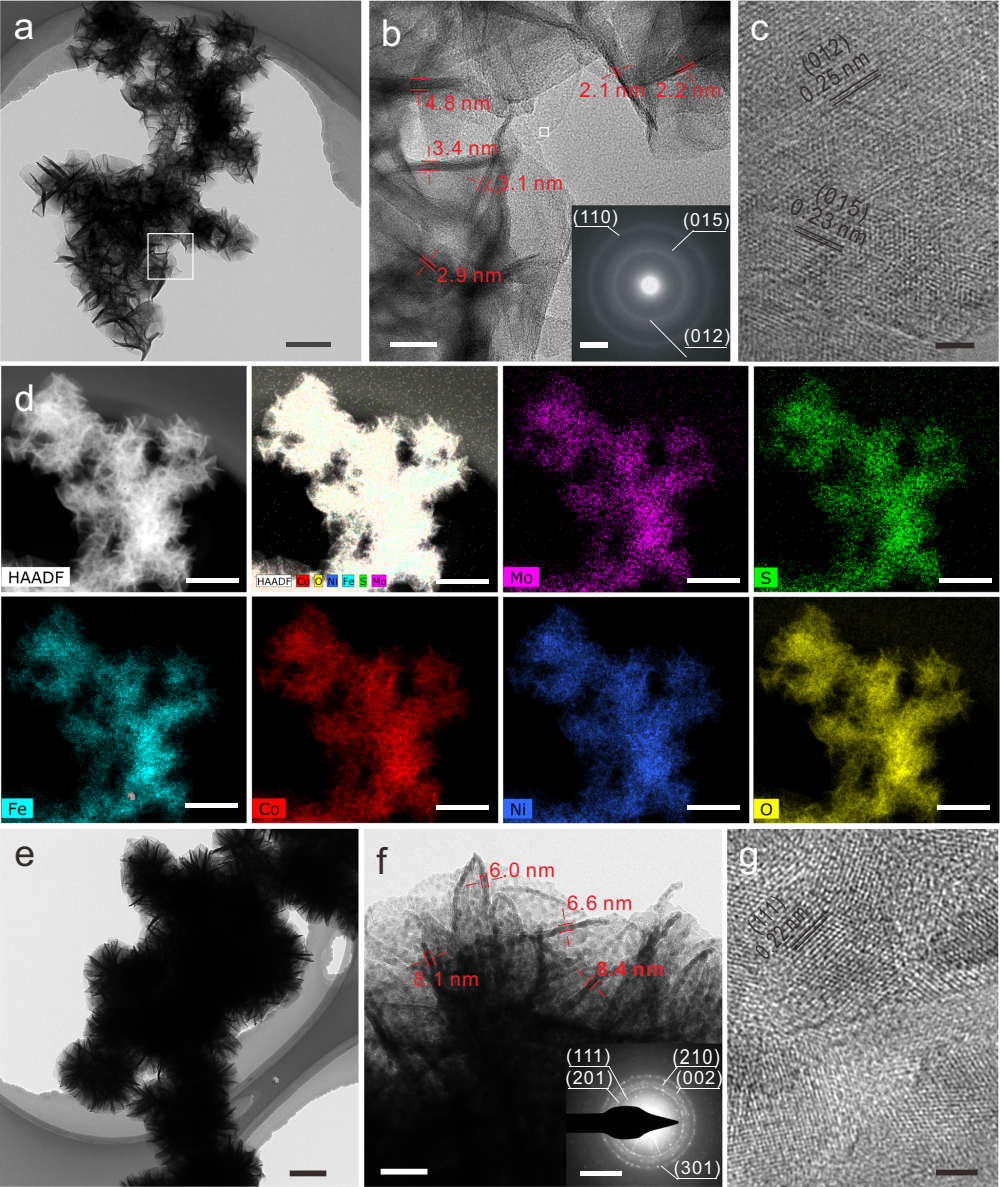
TEM characterization of different samples. **a**–**c** TEM images of MoS_2_/FeCoNi(OH)_x_. **d** Elemental mapping of MoS_2_/FeCoNi(OH)_x_. **e**–**g** TEM images of MoS_2_/FeCoNiP_x_. The insets in **b**, **f** are the corresponding SAED pattern. Scale bars: **a** 200 nm; **b** 20 nm; inset of **b** 5 1/nm; **c** 2 nm; **d** 200 nm; **e** 400 nm; **f** 40 nm; inset of **f** 5 1/nm; **g** 2 nm.

In order to further reveal the structure of different samples, transmission electron microscopy (TEM) and energy dispersive X-ray spectroscopy (EDS) analyses were carried out. The VGSs and MoS_2_ nanosheets for TEM characterization were detached from CF/VGSs and CF/VGSs/MoS_2_ by ultrasonication. Similar to our previous report^22^, the VGSs possess a tapered structure with the edges as thin as 1–2 atomic layers and inner part thicker (Supplementary Fig. 4). The fringe spacing was determined to be 0.35 nm, corresponding to the (002) plane of graphite. The SAED pattern shows the rings corresponding to (002), (101), (004), and (110) planes (Supplementary Fig. 4), confirming the graphitic structure of the VGSs. As for the MoS_2_ nanosheets (Supplementary Fig. 4), the spacing between the lattice fringes on the TEM images are 0.28 and 0.62 nm, corresponding to (100) and (002) planes of the hexagonal MoS_2_, respectively. The SAED pattern shows the rings corresponding to (002), (100), (106), and (110) planes (Supplementary Fig. 4), confirming the hexagonal structure of MoS_2_. Figure 3a shows the TEM image of MoS_2_/FeCoNi(OH)_x_ detached from the CF/VMFO by ultrasonication, which reveals about similar morphology to that shown in the SEM image (Fig. 2c). Figure 3b shows the TEM image taken from the white square in Fig. 3a, in which the thickness of the nanosheets was estimated to be 2.1–4.8 nm. The inset in Fig. 3b is the corresponding SAED pattern of the nanosheets, revealing that the sample is polycrystalline with poor crystallinity. The rings can be indexed to (012), (018), and (110) planes of rhombohedral NiFe(OH)_x_ or hexagonal NiCo(OH)_x_. The HRTEM image (Fig. 3c) taken from the white square in Fig. 3b exhibits fringe spacings of 0.25 and 0.23 nm, which correspond to (012) and (018) planes of rhombohedral NiFe(OH)_x_ or hexagonal NiCo(OH)_x_, respectively. It is indicated that the nanosheets shown in Fig. 3b are the composites of rhombohedral NiFe(OH)_x_ and hexagonal NiCo(OH)_x_. In order to exhibit the elemental distribution of MoS_2_ and FeCoNi(OH)_x_, high angle annular dark field scanning TEM (HAADF-STEM) and EDS mapping analysis were carried out. It is indicated that all the constituting elements of MoS_2_ and FeCoNi(OH)_x,_ i.e., Mo, S, Co, Fe, Ni, and O, are present and uniformly distributed (Fig. 3d). From the coexistance of all the constituting elements and their distribution ranges it is confirmed that the FeCoNi(OH)_x_ nanosheets are grown on the MoS_2_ nanosheets and firmly bonded each other. Further TEM analysis confirms that the FeCoNi(OH)_x_ nanosheets are grown on the MoS_2_ nanosheets, mainly on the side surface (Supplementary Fig. 5 and the text). Clearly, the areal density of the FeCoNi(OH)_x_ nanosheets can be greatly increased by growing on the MoS_2_ nanosheets than that growing on flat substrate surface, which will greatly increase the current density correspondingly. With respect to the CF/VMFP, the structure shown by the TEM is about similar to the CF/VMFO with the nanosheet structure well preserved (Fig. 3e, f). The thickness of the FeCoNiP_x_ nanosheets is 6.6–10.2 nm (Fig. 3f). The inset in Fig. 3f is the SAED pattern of the FeCoNiP_x_, which indicates that the FeCoNiP_x_ nanosheets also possess hexagonal structure. The rings on the SAED pattern correspond to the (111), (201), (210), (002), and (301) planes. Figure 3g shows the HRTEM image of the FeCoNiP_x_ nanosheets, where the lattice fringes with a spacing of 0.22 nm are presented. This interplanar spacing corresponds to (111) plane of the hexagonal Fe_2_P or Ni_2_P. The HAADF-STEM and EDS mapping analysis also demonstrate the presence and uniform distribution of Mo, S, Co, Fe, Ni, and P elements (Supplementary Fig. 6).

**Fig. 4:**
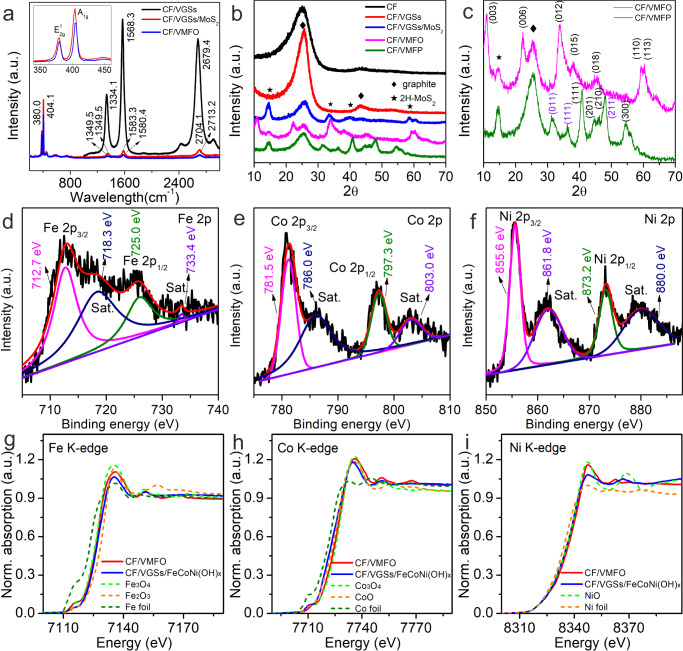
Raman spectra, XRD patterns, XPS spectra, and XANES spectra of different samples. **a** Raman spectra of CF/VGSs, CF/VGSs/MoS_2_, and CF/VMFO. **b** XRD patterns of different samples. **c** Magnified XRD patterns of CF/VMFO and CF/VMFP. **d**–**f** XPS spectra of Fe 2p (**d**), Co 2p (**e**), and Ni 2p (**f**) for CF/VMFO. **g**–**i** Normalized Fe (**g**), Co (**h**), and Ni (**i**) K-edge XANES and spectra of CF/VMFO, CF/VGSs/FeCoNi(OH)_x_, and standard samples. The inset of **a** is the magnified Raman spectra of CF/VGSs/MoS_2_ and CF/VMFO.

Figure 4a shows the Raman spectra of the CF/VGSs, CF/VGSs/MoS_2_, and CF/VMFO. The peaks at 1334, 1568, and 2680 cm^−1^ are ascribed to D, G, and 2D peaks of the VGSs, respectively. The ratio of I_G_/I_2D_ is ~1.2, indicating few layered structure of the VGSs, which is consistent with the TEM results. The peaks of the CF/VGSs/MoS_2_ and CF/VMFO at about 380.0 and 404.1 cm^−1^ are E_2g_^1^ and A_1g_ modes of 2H-MoS_2_, respectively. The separation between the E_2g_^1^ and A_1g_ modes is 24.1 cm^−1^, revealing few-layer feature of the MoS_2_ nanosheets^23,24^. This is consistent with the TEM results. X-ray diffraction (XRD) measurements were carried out to analyze the phase structure of the samples (Fig. 4b, c and Supplementary Fig. 7). The CF and CF/VGSs show diffraction peaks at ~25 and 42°, originated from the diffraction of (002) and (100) planes of graphite. There are seven diffraction peaks in the XRD pattern of the CF/VMFO. The peaks at ~15° and 25° represent the (002) plane of 2H-MoS_2_ and (002) plane of graphite, respectively. The remaining peaks correspond to the (003), (006), (012), (015), (018), and (110) crystal planes of NiFe(OH)_x_ (JCPDS Card no. 40-0215) and NiCo(OH)_x_ (40-0216), which is consistent with the TEM results. For the CF/VMFP, the diffraction peaks of the FeCoNi(OH)_x_ disappear and some different peaks appear. These peaks in Fig. 4c correspond to the (111), (201), (210), and (300) planes of Fe_2_P (JCPDS no. 51-0943) and Ni_2_P (JCPDS no. 03-0953). Others correspond to the (011), (111), and (211) planes of CoP (JCPDS no. 29-0497).

X-ray photoelectron spectroscopy (XPS) is used to further probe into elemental composition and bonding states of the CF/VMFO and CF/VMFP. The survey spectra shows presence of all the above elements in the CF/VMFO and CF/VMFP (Supplementary Fig. 8). The XPS peaks of Fe, Co, and Ni for CF/VMFO are shown in Fig. 4d–f. For the spectrum of Fe, the peaks at 712.7 and 725.0 eV correspond to Fe2p_3/2_ and Fe 2p_1/2_ binding energies with the satellite peaks at 718.3 and 733.4 eV, respectively, which is related to Fe^3+^^25^. For Co, the peaks at 781.5 and 797.3 eV along with two satellite peaks at 786.0 and 803.0 eV arise from Co^2+^^13^. As for the spectrum of Ni, the peaks located at 855.6 and 873.2 eV with two satellite peaks at 861.8 and 880.0 eV are assigned to Ni 2p_3/2_ and Ni 2p_1/2_ of Ni^2+^^26^. All these features reveal that Fe, Co, and Ni are present in the form of Fe^3+^, Co^2+^, and Ni^2+^. The binding energies of Fe 2p, Co 2p, and Ni 2p in the CF/VMFO increase slightly compared with the CF/VGSs/FeCoNi(OH)_x_ (Supplementary Fig. 9), indicating that Fe, Co, and Ni in CF/VMFO are in higher valence state, which is beneficial to the OER activity^27^. These results suggest that there exist electronic interactions between MoS_2_ and FeCoNi(OH)_x_, indicative of the presence of the electronic coupled interface between them. As for the spectra of O, the peaks at 530.5, 531.4, 532.1, and 532.7 eV originate from oxide species, oxygen ions in FeCoNi(OH)_x_, defects with low oxygen coordination, and physic-/chemisorbed water on the surface, respectively (Supplementary Fig. 10)^28–30^. The defects with low oxygen coordination possess high capability to adsorb reaction intermediates during the OER reaction. Electron spin resonance (ESR) spectra were measured to further confirm the presence of the O defects in FeCoNi(OH)_x_. The ESR spectrum of the CF/VMFO shows a pair of steep peaks with a symmetric distribution and *g* = 2.003 (Supplementary Fig. 11)^31,32^, meaning electron trapping at oxygen vacancies in FeCoNi(OH)_x_. This is consistent with the reports about the presence of oxygen vacancies^31,32^. With respect to the CF/VMFP, the bonding states of Fe, Co, Ni, and P elements were also analyzed by their respective XPS spectra (Supplementary Fig. 12). For the Fe 2p spectrum, the peaks at 711.5/723.6 eV and 715.6/727.3 eV are attributed to Fe^2+^ and Fe^3+^, respectively^18^. For the Co 2p spectrum, the two peaks at 778.0 and 792.8 eV result from partial oxidation of Co atoms (Co^δ+^, δ is likely close to 0). The two satellite peaks at 785.9 and 802.6 eV as well as the peaks at 781.2 and 797.7 eV arise from the oxidized Co (Co^3+^) from CoP^33^. The peaks at 853.6, 856.4, and 861.0 eV for the Ni 2p spectrum correspond to Ni_2_P, Ni-PO_x_, and the corresponding satellite peak, respectively^19^. For the spectra of P, the peaks at 129.3 and 130.1 eV originate from phosphorus anions of metal phosphides, and those at 133.5 and 134.3 eV originate from phosphate-like P due to possible surface oxidation^20^. By using inductively coupled plasma mass spectrometer (ICP-MS), the molar ratio of Fe, Co, and Ni in FeCoNi(OH)x has been determined to be 1:1.5:1.2 and that of Fe, Co, Ni, and P in FeCoNiPx is 1:1.4:1.1:2.6. Furthermore, X-ray absorption fine structure (XAFS) spectroscopy measurements at the Fe, Co, and Ni K-edges were conducted to investigate the composition and valence state of CF/VMFO. Figure 4g–i shows the X-ray absorption near edge structure (XANES) for Fe, Co, and Ni K-edges of CF/VGSs/FeCoNi(OH)_x_, CF/VMFO, and various standard samples, respectively. Compared with CF/VGSs/FeCoNi(OH)_x_, the CF/VMFO shows increased intensities at Fe, Co, and Ni K-edge XANES. This indicates the electron transfer from Fe, Co, and Ni atoms to neighboring atoms (O, Mo, and S) because of the coupling effects in the composite materials^34,35^, resulting in reduced electron density of Fe, Co, and Ni atoms in CF/VMFO. This result is consistent with that obtained by XPS.

### The OER electrocatalysis

**Fig. 5:**
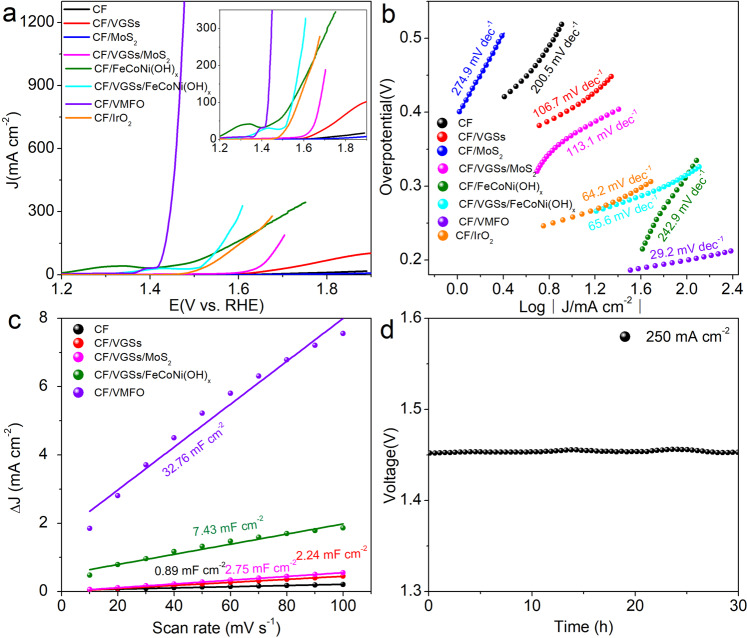
OER performance of different samples tested in 1 M KOH. **a** LSV curves. **b** Tafel plots. **c** Capacitive currents at different scan rates. **d** Time-dependent overpotential curve of CF/VMFO at 250 and 500 mA cm^−2^.

The OER performance of the catalysts was investigated in 1.0 M KOH solution in a three-electrode system. The polarization curves of different samples are shown in Fig. 5a, it is obvious that CF/VMFO has the best OER performance among the samples. The overpotential at the current densities of 500 and 1000 mA cm^−2^ are only 225 and 241 mV, respectively (see uncorrected curve in Supplementary Fig. 13). The CF/VMFO shows a high current density of 1297 mA cm^−2^ at the overpotential of 250 mV. This is not only superior to the commercial IrO_2_ on the carbon felt (CF/IrO_2_), but also exceeds the performance of the reported OER catalysts^10,12,14,16^. A small peak appears before the rising straight line on the polarization curves of the CF/VMFO, CF/VGSs/FeCoNi(OH)_x_, and CF/FeCoNi(OH)_x_, which is related to the oxidation of Ni or Co^36–38^. In contrast, for the CF, CF/VGSs, CF/MoS_2_, and CF/VGSs/MoS_2_ this oxidation peak is not observed. The polarization curves of the CF/FeCoNi(OH)_x_ and CF/VGSs/FeCoNi(OH)_x_ show much inferior catalytic activity to the CF/VMFO. Obviously, the high catalytic activity of the CF/VMFO arises from FeCoNi(OH)_x_, which may result from the strong coupling effect among VGSs, FeCoNi(OH)_x_, and MoS_2_. This can be further manifested by the Tafel slope of the different samples. Tafel slope is able to evaluate catalytic kinetics of reactions^39–41^. As shown in Fig. 5b, the Tafel slopes of CF/VMFO is 29.2 mV dec^−1^, which is much smaller than those of the CF (200.5 mV dec^−1^), CF/VGSs (106.7 mV dec^−1^), CF/MoS_2_ (274.9 mV dec^−1^), CF/VGSs/MoS_2_ (101.7 mV dec^−1^), CF/FeCoNi(OH)_x_ (242.9 mV dec^−1^), CF/VGSs/FeCoNi(OH)_x_ (65.6 mV dec^−1^), and CF/IrO_2_ (64.2 mV dec^−1^).

Previously, many electrocatalysts have been prepared for OER and great progress has been achieved. For example, Yu et al. prepared Cu nanowires on Cu foam and then few-layered NiFe LDH was electrodeposited on the Cu nanowires (Cu@NiFe LDH/CF)^10^. The obtained catalyst showed a current density of about 40 mA cm^−2^ at an overpotential of 250 mV. Hou et al. have used exfoliated graphene (EG) as substrate to grow Co_0.85_Se and NiFe-LDH sequentially via two steps of hydrothermal treatments^14^. The obtained EG/Co_0.85_Se/NiFe-LDH exhibited a current density of 80 mA cm^−2^ at an overpotential of 250 mV with the Tafel slope of 57 mV dec^−1^. Yu et al. fabricated FeP/Ni_2_P on Ni foam by two times of immersion in Fe(NO_3_)_3_ solution and subsequent phosphidation^19^. The obtained product presented an overpotential of about 252 mV at 300 mA cm^−2^ with a small Tafel slope of 22.7 mV dec^−1^. Liang et al. prepared NiFe^−^LDH on Ni foam via hydrothermal method, and then converted it to NiFeP through PH_3_ treatment in plasma, later NiFe^−^OH was electrodeposited on the NiFeP (NiFe^−^OH/NiFeP/NF)^12^. This sample showed a current density of about 290 mA cm^−2^ at an overpotential of 250 mV with the Tafel slope of 39 mV dec^−1^.

It can be seen that the present CF/VMFO possesses excellent catalytic performance towards OER with smaller overpotential and Tafel slope than most of the previous reports (Supplementary Table 1). Particularly, the current density of the CF/VMFO is much higher than other reported catalysts, which is highly required for practical application. The combination of the VGSs, MoS_2_, and FeCoNi(OH)_x_ generates excellent catalytic performance although any of them behaves not well individually. Clearly, the excellent performance of the CF/VMFO results from the synergistic effect of the individual components, i.e., VGSs, MoS_2_, and FeCoNi(OH)_x_. First of all, as calculated below, the combination of different components changes the electronic structure of the catalysts, which provides easier path for the electron transfer. In addition, the multilayer-stacked nanosheet structure makes the MoS_2_ and FeCoNi(OH)_x_ nanosheets suspended and fully immersed in the electrolyte solution, which makes the electrolyte solution flow more freely without dead space around the MoS_2_ and FeCoNi(OH)_x_ nanosheets and is thus beneficial to the transfer and access of the electrolyte ions. In particular, as shown in Fig. 2c and Supplementary Fig. 5, the FeCoNi(OH)_x_ nanosheets grow on the side surface of the MoS_2_ nanosheets, which makes the area density of the FeCoNi(OH)_x_ nanosheets increase greatly. This may be the important reason for the high current density. Furthermore, the VGSs possess higher conductivity than the CFs, which provide faster electron transfer path for the reactions. Although the FeCoNi(OH)_x_ nanosheets may be the dominant contributor to the high catalytic activity of the CF/VMFO, the role of the MoS_2_ nanosheets is especially key. The strategy to improve the performance of electrocatalysts by integrating different materials has been widely used^14,42–45^. For example, Hui et al. prepared iron–cobalt LDH (ICLDH) nanosheet arrays on nickel foam (NF) by hydrothermal treatment and then used hexaethynylbenzene to intercalate into the interlayer space of the iron–cobalt LDH^42^. After polymerization reaction, the hexaethynylbenzene converted into graphdiyne. Along with the formation of graphdiyne, the original LDH nanosheets exfoliated further, forming graphdiyne-coated ICLDH nanosheets with a sandwich structure. The graphdiyne-coated ICLDH nanosheets possess excellent OER performance with small overpotentials of 275 and 278 mV at current densities of 500 and 1000 mA cm^−2^, respectively. Other structures reported include vertical MoS_2_ nanosheets on hydrogenated graphene^44^, NiCo-LDH on MoS_2_ nanosheets with vertical orientation^43^, NiFe-LDH nanosheets on MXene^45^, NiFe-LDH nanosheets on CoFe-LDH nanosheets^46^, and Co_0.85_Se/NiFe-LDH nanosheets on exfoliated graphene foil^14^, etc. These catalysts are mostly prepared by hydrothermal method. Comparing with the previous reports as described above, the present structure of vertical nanosheets on vertical nanosheets with three layers is unique and has not been reported so far. Besides increasing conductivity and tuning electronic structure, the present three-layer structure allows the electrolyte solution flow more freely and thus increase transfer rate of the electrolyte ions. In addition, comparing with the widely used hydrothermal method, the methods of thermal CVD and electrodeposition used in this work have been well established industrically, ensuring easy scale-up production of the present electrocatalysts.

In order to further understand the roles of different components in the composites, electrochemically active surface area (ECSA) and electrochemical impedance spectra (EIS) were measured for the different samples. Higher ECSA implies more active sites for catalytic reactions and is good for water molecule adsorption and intimate contact with the electrolyte^10^. ECSA is proportional to the double layer capacitance (C_dl_)^12,47^. Figure 5c presents the C_dl_ calculated from the corresponding CV curves (Supplementary Fig. 14). The C_dl_ of the CF, CF/VGSs, CF/VGSs/MoS_2_, CF/VGSs/FeCoNi(OH)_x_, and CF/VMFO are 0.9, 2.2, 2.8, 7.4, and 32.8 mF cm^−2^, respectively. It is found that the combination of VGSs, MoS_2_, and FeCoNi(OH)_x_ nanosheets could greatly increase the ECSA. We consider that the suspended structure and changes in surface property due to introduction of MoS_2_ account for the high ECSA of the CF/VMFO, and thus contributing to the enhancement of the OER performance. EIS could reflect the OER kinetics of the samples. It is observed that there exist two semicircles in the EIS spectra of all the samples (Supplementary Fig. 15). The semicircle in the high frequency region is related to charge transfer resistance and the double layer capacitance, while the low frequency one is related to the adsorption of reaction intermediate (HO*, O*, and HOO*) and the OER taking place at the electrode–electrolyte interface during OER process^48,49^. The high frequency semicircle of the CF/VMFO has the smallest diameter among the different samples, indicating that the charge transfer process could be enhanced by the combination of the VGSs, MoS_2_, and FeCoNi(OH)_x_ nanosheets. The low frequency semicircle diameter decreases gradually in the order of CF/VGSs/MoS_2_, CF/VGSs/FeCoNi(OH)_x_, and CF/VMFO, which indicates that more active sites with higher activity for absorption of the reaction intermediate could be provided by the combination of the VGSs, MoS_2_, and FeCoNi(OH)_x_ nanosheets. Durability is an important index for the practical application of catalysts. Figure 5d shows the chronopotentiometry curve of the CF/VMFO at a current density of 250 and 500 mA cm^−2^ in 1 M KOH solution. The CF/VMFO has excellent stability with a voltage of 1.45 and 1.52 V at 250 and 500 mA cm^−2^ after testing for 30 h, respectively. The morphology of CF/VMFO after stability test remains unchanged and the multilayer-stacked structure is not damaged (Supplementary Fig. 16). This excellent stability may be caused by the low overpotential, high chemical stability, and high mechanical stability of the catalyst materials, which mainly originate from their unique structure. It should be pointed out that the graphene is easily oxidized at high potentials, which will bring about stability problem during operation. The stability test shown in Fig. 5d suggests that the present VGSs are stable, which may be because the VGSs possess high crystallinity due to the high growth temperature of 1200 °C and multi-atomic-layer thickness in the inner part.

The above results indicate that the catalytic activity could be greatly enhanced by introducing MoS_2_ into the system. In order to explore the catalytic mechanism, we calculated the Gibbs free energy of each reaction stage and overpotential by DFT + U for OER based on the 4e^−^ mechanism proposed by Norskov for water oxidation as follows.

1 $$\ast + {\mathrm{H}}_{\mathrm{2}}{\mathrm{O}} \to \ast {\mathrm{OH}} + \left( {{\mathrm{H}}^ + + {\mathrm{e}}^ - } \right)\;\;\;{\Delta}{G}_{\mathrm{I}}$$

2 $$\ast {\mathrm{OH}} \to \ast {\mathrm{O}} + \left( {{\mathrm{H}}^ + + {\mathrm{e}}^ - } \right)\;\;\;{\Delta}{G}_{{\mathrm{II}}}$$

3 $$\ast {\mathrm{O}} + {\mathrm{H}}_2{\mathrm{O}} \to \ast {\mathrm{OOH}} + \left( {{\mathrm{H}}^ + + {\mathrm{e}}^ - } \right)\;\;\;{\Delta}{G}_{{\mathrm{III}}}$$

4 $$\ast {\mathrm{OOH}} \to \ast + {\mathrm{O}}_2 + \left( {{\mathrm{H}}^ + + {\mathrm{e}}^ - } \right)\;\;\;{\Delta}{G}_{{\mathrm{IV}}}$$

Where * represents the active sites on catalysts, Δ*G*_I_, Δ*G*_II_, Δ*G*_III_, and Δ*G*_IV_ represent the Gibbs free energy changes of the reaction steps.

The overpotential in OER is calculated as follows:

5 $${\upeta}_{{\mathrm{OER}}} = \max \left\{ {{\Delta}{G}_{\mathrm{I}},{\Delta}{G}_{{\mathrm{II}}},{\Delta}{G}_{{\mathrm{III}}},{\Delta}{G}_{{\mathrm{IV}}}} \right\}/{\mathrm{e}} - {\mathrm{1}}{\mathrm{.23}}$$

**Fig. 6:**
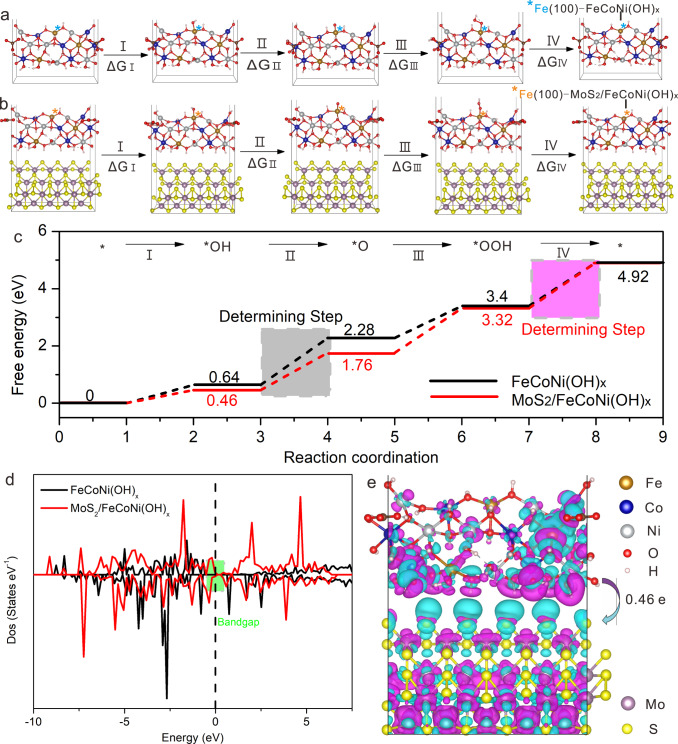
Theoretical calculations of the electrocatalysts. **a** Proposed 4e^-^ mechanism of OER on FeCoNi(OH)_x_ (see panel **e** to distinguish different atoms). **b** Proposed 4e^-^ mechanism of OER on MoS_2_/FeCoNi(OH)_x_ (see panel **e** to distinguish different atoms). **c** Gibbs free energy diagram for the four steps of OER on FeCoNi(OH)x and MoS_2_/FeCoNi(OH)_x_ at the position of Fe ions. **d** Total density of states curves of FeCoNi(OH)_x_ and MoS_2_/FeCoNi(OH)_x_. **e** Differential charge density between FeCoNi(OH)_x_ and MoS_2_. The Fe ion (*) in **a**, **b** is the active site.

The 4e^−^ mechanism of OER of FeCoNi(OH)_x_ and MoS_2_/FeCoNi(OH)_x_ at the positions of Fe ions in the (100) planes is shown in Fig. 6a, b. Supplementary Fig. 17 is the structural model before and after optimization of the compositing structure. Figure 6c shows the free energy of the reaction intermediates after each reaction step. Supplementary Table 2 lists the free energy changes of each reaction step at different sites and the OER overpotential for FeCoNi(OH)_x_ and MoS_2_/FeCoNi(OH)_x_. The rate determining step for Fe sites on FeCoNi(OH)_x_ is the second step (Δ*G*_II_ = 1.64 eV), i.e., *OH ➔ *O. The rate determining step of the Fe sites in MoS_2_/FeCoNi(OH)_x_ changes to the fourth step with the energy change of Δ*G*_IV_ = 1.60 eV, which is smaller than that of FeCoNi(OH)_x_. This is because the charge of FeCoNi(OH)_x_ is redistributed after compositing with MoS_2_, which causes the change of the rate determining step and decrease of the overpotential. The overpotential of FeCoNi(OH)_x_ and MoS_2_/FeCoNi(OH)_x_ is 0.41 and 0.37 V at the positions of Fe, respectively, indicating that the OER is easier for MoS_2_/FeCoNi(OH)x. Moreover, the overpotential of MoS_2_/FeCoNi(OH)_x_ is 0.91 and 1.04 V at the positions of Co and Ni ions on (100) planes, respectively (Supplementary Fig. 18). Therefore, Fe ions play a key role in OER of MoS_2_/FeCoNi(OH)_x_ and the Fe ions on (100) planes are active sites^50^. It is because the abundant unsaturated coordinated Fe ions in the edges of FeCoNi(OH)_x_ nanosheets contribute greatly to the OER. It is worth noting that the calculated overpotential difference of 40 mV for the FeCoNi(OH)_x_ and MoS_2_/FeCoNi(OH)_x_ is close to their experimental onset-potential difference of 73 mV, which is obtained from their LSV curves shown in Fig. 5a.

In order to further understand the catalytic performance of MoS_2_/FeCoNi(OH)_x_, the electronic structures were calculated by DFT + U. Figure 6d is the total densities of the states (DOS) of FeCoNi(OH)x and MoS_2_/FeCoNi(OH)_x_. It is found that the bandgap of MoS_2_/FeCoNi(OH)_x_ is smaller than that of FeCoNi(OH)_x_, indicating that the electron transfer barrier decreases and the conductivity increases after compositing. Comparing with the total DOS of FeCoNi(OH)_x_, the local DOS of FeCoNi(OH)_x_ in MoS_2_/FeCoNi(OH)_x_ changes not much, especially near Fermi level (Supplementary Fig. 19), suggesting that the smaller bandgap of MoS_2_/FeCoNi(OH)_x_ may be just caused by the metallic edges of MoS_2_. This is consistent with the energy band structures of FeCoNi(OH)_x_ and MoS_2_/FeCoNi(OH)_x_ in Supplementary Fig. 20. FeCoNi(OH)_x_ has obvious forbidden bands, which makes the electron transfer more difficult, while the band gap of MoS_2_/FeCoNi(OH)x is almost negligible, which is highly advantageous to charge transfer. Figure 6e shows the differential charge density between FeCoNi(OH)_x_ and MoS_2_. The magenta and cyan regions represent charge depletion and accumulation ones, respectively. Obvious electron redistribution is observed at the interface of MoS_2_/FeCoNi(OH)_x_. The Bader charges on Fe, Co, and Ni sites are 1.33, 1.09, and 0.66 | e | , respectively. Bader charge analysis indicates that about 0.46 electrons per supercell are transferred from FeCoNi(OH)_x_ to MoS_2_ at the interface. The electron transfer direction is consistent with the XANES results. The XPS measurements also suggest that Fe, Co, and Ni in CF/VMFO are in higher valence state (Supplementary Fig. 9). The electron transfer results in hole aggregation on FeCoNi(OH)_x_, which is beneficial to obtain the optimal value of binding energies for the intermediates of OER on the catalysts^51^. The calculated binding energies of MoS_2_/FeCoNi(OH)_x_ with the intermediates for OER are smaller than those of FeCoNi(OH)_x_ (Supplementary Table 3), which is favorable for the next step reaction and desorption of the product. The above calculated results confirm that the compositing of FeCoNi(OH)_x_ with MoS_2_ could increase the electrocatalytic activity by tailoring the electronic structure.

### The HER electrocatalysis and overall water splitting

**Fig. 7:**
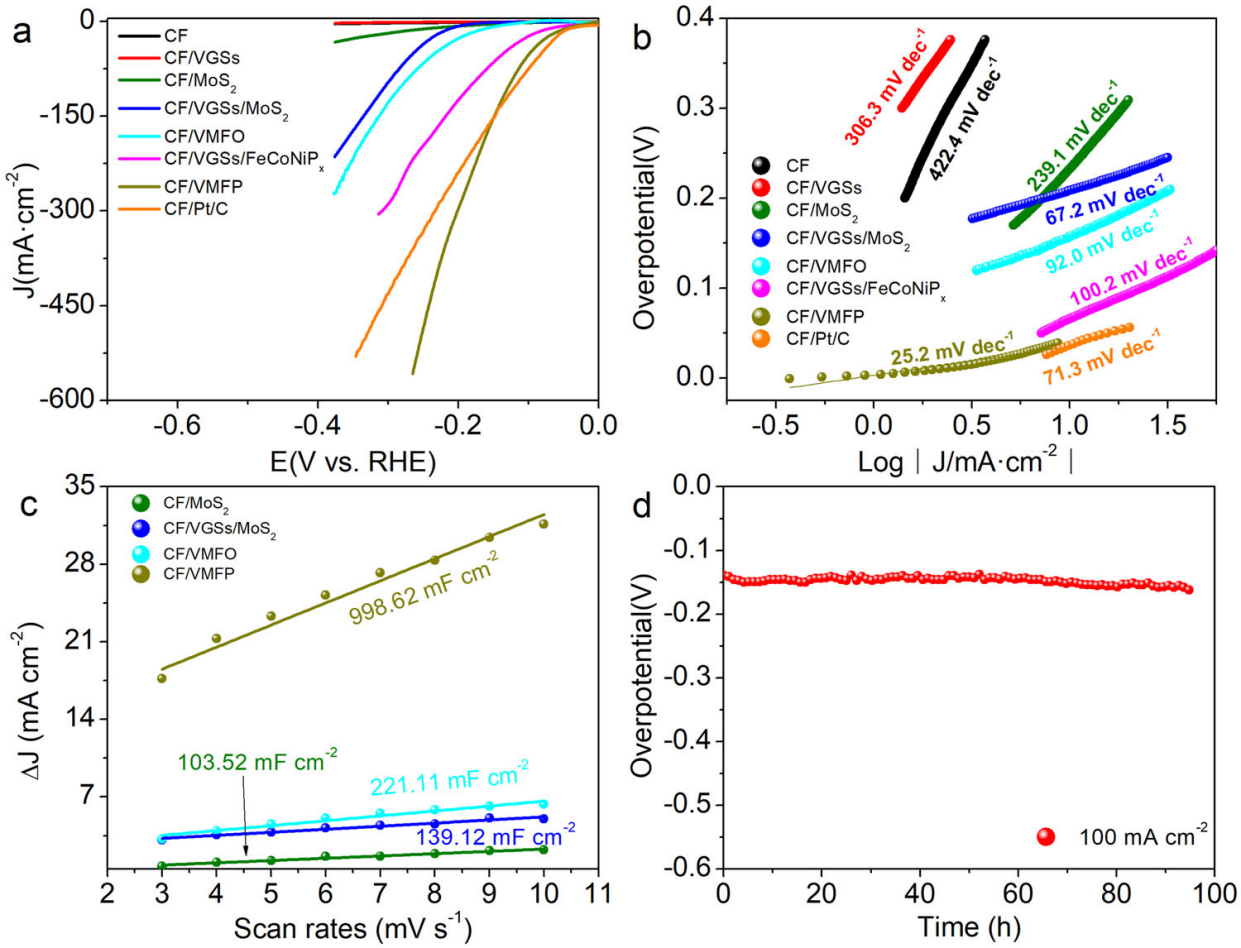
HER performance of different samples tested in 1 M KOH. **a** LSV curves. **b** Tafel plots. **c** Capacitive currents at different scan rates. **d** Time-dependent overpotential curves of CF/VMFP at 100 mA/cm^2^.

The electrocatalytic performance of different samples for HER was also measured. As shown in Fig. 7a, b, among the different samples including CF/Pt/C, the HER performance of the CF/VMFP is the best. The overpotentials of this sample at the current densities of 10 and 100 mA cm^−2^ are 43 and 127 mV, respectively (see uncorrected curve in Supplementary Fig. 21), and the Tafel slope is 25.2 mV dec^−1^. In contrast, the CF/VMFO before being phosphatized exhibits an overpotential of 157 mV at 10 mA cm^−2^ and Tafel slope of 92.0 mV dec^−1^. However, the CF and CF/VGSs show no electrocatalytic activity for HER. The CF/MoS_2_ and CF/VGSs/MoS_2_ possess some HER electrocatalytic activity, but much inferior to the CF/VMFP. The overpotentials of the CF/MoS_2_ and CF/VGSs/MoS_2_ are 232 and 209 mV at 10 mA cm^−2^, respectively. The HER electrocatalytic activity has been reported previously for the MoS_2_^16,17^, which is limited by its poor conductivity. Although the overpetential of CF/VGSs/MoS_2_ decreases a little at 10 mA cm^−2^ in contrast to CF/MoS_2_, the Tafel slope decreases from 239.1 to 67.2 mV dec^−1^, indicating faster kinetic process of the CF/VGSs/MoS_2_ than CF/MoS_2_ because of the improved conductivity of CF/VGSs/MoS_2_. The CF/VGSs/FeCoNiP_x_ exhibits an overpotential of 66 mV at 10 mA cm^−2^ and Tafel slope of 100.2 mV dec^−1^. Clearly, the high electrocatalytic activity of the CF/VMFP is mainly from the FeCoNiP_x_ nanosheets. This HER performance ranks among the top of the reported ones^15,19,52–55^. For example, the CF/VMFP (*j*_150 mV_ = 140 mA cm^−2^) is superior to MoS_2_/MoC (*j*_150_ _mV_ = 120 mA cm^−2^)^52^, Ni-Fe/TiN/CC (*j*_150_ _mV_ = 60 mA cm^−2^)^53^, Fe-CoP/NF (*j*_150_ _mV_ = 48 mA cm^−2^)^15^, and S:CoP@NF (*j*_150_ _mV_ = 33 mA cm^−2^) and comparable to FeP/Ni_2_P (*j*_150_ _mV_ =140 mA cm^−2^)^19^ and holy NiCoP NS (*j*_150_ _mV_ = 150 mA cm^−2^)^55^ (Supplementary Table 4). Similar to the OER performance, the excellent HER performance of the CF/VMFP should result from the synergistic effect of the individual components, i.e., VGSs, MoS_2_, and FeCoNiP_x_. Of course, the HER performance is mainly from the FeCoNiP_x_ nanosheets, but the role of MoS_2_ nanosheets is crucial in the high catalytic activity of the CF/VMFP.

The ECSA was further measured for the different samples to probe into their electrocatalytic performance. As shown in Fig. 7c, the *C*_dl_ of CF/MoS_2_, CF/VGSs/MoS_2_, CF/VMFO, and CF/VMFP are 103.5, 139.1, 221.1, and 998.6 mF cm^−2^, respectively, which were calculated from the corresponding CV curves (Supplementary Fig. 22). Clearly, the metal phosphides exceed the other components in ECSA. It is observed that the *C*_dl_ of the same catalysts such as CF/VMFO and CF/VGSs/MoS_2_ for OER and HER is very different, which is clearly because the reaction mechanism is different and the active sites of a catalyst for the adsorption of different ions and intermediates are different at different potential ranges^19^. In addition, the CF/VMFP has excellent stability. As shown in Fig. 7d, the overpotential only increased from 140 mV to 150 mV after testing for 95 h at 100 mA cm^−2^.

**Fig. 8:**
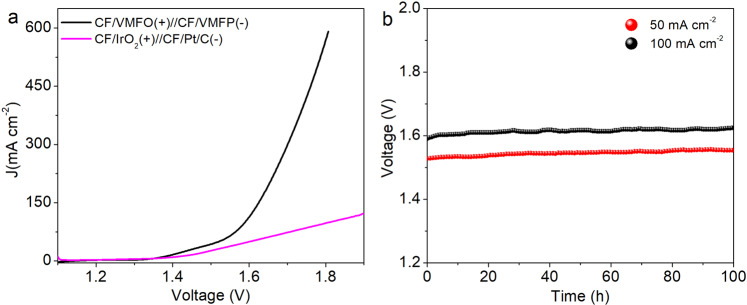
Overall water splitting activity of the samples in 1 M KOH solution. **a** Polarization curves of CF/VMFO(+)//CF/VMFP(−) and CF/IrO_2_//CF/Pt/C at a scan rate of 2 mV s^−1^. **b** Catalytic stability of CF/VMFO(+)//CF/VMFP(−) at 50 and 100 mA cm^−2^ tested in a two-electrode configuration.

Based on the above results, overall water splitting performance of the samples was tested by using the CF/VMFO and CF/VMFP as anode and cathode, respectively. For comparison, CF/IrO_2_(+)//CF/Pt/C(−) was also tested in 1 M KOH solution. Figure 8a shows the polarization curves of the above two water splitting electrolyzers. It is indicated that for producing the current densities of 10, 50, and 100 mA cm^−2^ the voltages of 1.37, 1.52, and 1.59 V are required for the CF/VMFO(+)//CF/VMFP(−) cell (see uncorrected curve in Supplementary Fig. 23), while the voltages of 1.40, 1.60, and 1.81 V are required for the CF/IrO_2_(+)//CF/Pt/C(−) cell. Clearly, the overall water splitting performance of our samples is better than that of CF/IrO_2_(+)//CF/Pt/C(−), especially at high current density. The water splitting potential of 1.59 V at the current density of 100 mA cm^−2^ is smaller than those of most reported electrocatalysts, such as Cu@NiFe LDH(+)//Cu@NiFe LDH(−) (1.69 V)^10^, Ni_2_P-Ni_3_S_2_ HNAs/NF(+)//Ni_2_P-Ni_3_S_2_ HNAs/NF(−) (1.6 V)^56^, NiFeOx(+)//NiFe-P(−) (1.76 V)^57^, MoNi_4_(+)/MoS_2_//Ni_3_S_2_(−) (1.67 V)^58^, and Cu@NiFe LDH(+)//Ni_2(1-x)_Mo_2x_p(−) (1.65 V)^59^ (Supplementary Table 5). Currently, most of the water splitting electrocatalysts reported require voltages higher than 1.62 V to reach 100 mA cm^−2^. It should be noticed that our OER electrocatalyst CF/VMFO is much better than the HER electrocatalyst CF/VMFP. We believe that the overall water splitting performance could be further improved if using better HER electrocatalyst. The working stability of our electrocatalysts during overall water splitting was tested (Fig. 8b), which indicates that the voltage keeps almost unchanged after 100 h at the current densities of both 50 and 100 mA cm^−2^, indicating excellent stability.

## Discussion

In summary, multilayer-stacked nanosheet composites were prepared by growing the VGSs, MoS_2_, and FeCoNi(OH)_x_ or FeCoNiP_x_ nanosheets on CFs successively. The stacked structure of nanosheets on nanosheets has the advantages of easier transfer and access of electrolyte ions, higher areal density of the nanosheets, and improved electronic structure of the catalysts. The combination of the VGSs, MoS_2_, and FeCoNi(OH)_x_ or FeCoNiP_x_ nanosheets in this way generates remarkable synergistic effect and thus results in good catalytic performance towards OER and HER including small overpotential, small Tafel slope, and high stability. Besides the structure of stacked vertical nanosheets, the intermediate MoS_2_ nanosheets play a key role in the electrocatalytic process possibly by lowering the energy barrier for the electron transfer between the FeCoNi(OH)x or FeCoNiP_x_ nanosheets and VGSs. With respect to the OER, theoretical calculation confirms that the compositing of FeCoNi(OH)_x_ with MoS_2_ could generate favorable electronic structure and decrease the OER overpotential, accounting for the improvement of the electrocatalytic activity. When using the CF/VMFO and CF/VMFP as anode and cathode for overall water splitting, a current density of 100 mA cm^−2^ was achieved at 1.59 V, which keeps almost unchanged during 100 h testing. The present electrocatalysts for water splitting are of high potential for practical application.

## Methods

### Preparation of CF/VGSs

The CF/VGSs were prepared by growing vertical graphene sheets on carbon felt in a tube furnace at 1200 °C from the mixture of CH_4_ and H_2_, where the flow rates of CH_4_ and H_2_ are 6 and 160 sccm, respectively.

### Preparation of CF/VGSs/MoS_2_

MoCl_5_ (99.99%, anhydrous, Sigma-Aldrich) and sulfur (99.99%, Sigma-Aldrich) were used as the precursors to prepare the MoS_2_ nanosheets. During experiments MoCl_5_ was weighed in an argon-filled glove box to avoid hydrolysis in air while sulfur was weighed in air. 30 mg of MoCl_5_, 0.15 g of sulfur powder, and CF/VGSs were placed in different crucibles in an argon-filled glove box, which were put in a glass tube sleeve sealed with sealant. The CF/VGSs was placed in one end while the MoCl_5_ and sulfur crucible were placed 12 and 5 cm away from the CF/VGSs. Then the glass tube sleeve was taken out from the glove box and the sealant was peeled off, then quickly transferred to the tube furnace. The tube furnace was first evacuated to a base pressure of 10 mTorr and purged twice with Ar. Then the tube furnace was heated to 500 °C under Ar atmosphere with a flow rate of 50 sccm. The heating rate is 20 °C/min and the reaction time is 10 min.

### Preparation of CF/VMFO

Nickel nitrate (Ni(NO_3_)_2_·6H_2_O, 98%, Macleans), cobalt nitrate(Co(NO_3_)_2_·6H_2_O, 97%, Aladdin), and ferric nitrate(Fe(NO_3_)_3_·9H_2_O, 98.5%, Macleans) were used as the precursors to prepare the FeCoNi(OH)_x_ nanosheets. The FeCoNi(OH)_x_ nanosheets were electrodeposited on MoS_2_ nanosheets in a standard three-electrode electrochemical system, where the CF/VGSs/MoS_2_, Pt foil, and Ag/AgCl were used as the working, counter, and reference electrode, respectively. The aqueous solution with 0.1 M Ni(NO_3_)_2_, 0.1 M Co(NO_3_)_2_, and 0.05 M Fe(NO_3_)_3_ was used as the electrodepositing solution. The electrodeposition was carried out for 200 s at a constant cathode voltage of −1.0 V. After electrodeposition, the obtained composite electrode was washed several times with deionized water and ethanol, and dried at 70 °C in the air. For comparison, FeCoNi(OH)_x_ nanosheets were also directly electrodeposited on the carbon felt following the above stated conditions. The mass loading of VGSs, MoS_2_, FeCoNi(OH)_x_, and FeCoNiP_x_ were determined by weighing the samples before and after growth. The mass loading of VGSs, MoS_2_, and FeCoNi(OH)_x_ in the CF/VMFO is about 1.1, 1.7, and 6.7 mg/cm^2^.

### Preparation of CF/VMFP

The CF/VMFP were prepared by converting the FeCoNi(OH)_x_ nanosheets into NiCoFeP_x_ nanosheets. Firstly, a piece of the CF/VMFO with a size of 3 × 4 cm^2^ was placed in the center of the tube furnace and 200 mg of sodium hypophosphite (NaH_2_PO_2_) was placed upstream of the CF/VMFO. Then the furnace was heated at 280 °C for 3 h at a heating rate of 2 °C/min in an argon stream. The mass loading of FeCoNiP_x_ in the CF/VMFP is about 5.9 mg/cm^2^.

### Electrochemical measurements

All electrochemical measurements were carried out on an electrochemical workstation (CHI760) with a three-electrode system in 1 M KOH solution. During measurements, the prepared samples were used as the working electrodes and a graphite rod and Hg/HgO electrode were used as the counter and reference electrode, respectively. All the potentials were transformed to those relative to RHE using the following equations: *E* (RHE) = *E*_Hg/HgO_ + 0.098 + 0.059 pH. The OER tests were performed in O_2_-saturated 1 M KOH solution. The HER tests were performed in N_2_-saturated 1 M KOH solution. Polarization curves were obtained using linear sweep voltammetry (LSV) at a scan rate of 2 mV s^−1^ corrected by 90% iR compensation. Cyclic voltammetry (CV) curves were collected at different scan rates in the potential range of 1.025–1.125 V vs. RHE to evaluate the double-layer capacitance values for OER and from 0.05 V to 0.15 V vs. RHE for HER. Electrochemical impedance spectroscopy (EIS) was carried out at an overpotential of 300 mV from 0.1 Hz to 100 kHz with an amplitude of 10 mV. The overall water splitting performance was evaluated in 1 M KOH using a two-electrode configuration, and the polarization curve was recorded at a scan rate of 2 mV s^−1^. For the comparison experiment, Pt/C and IrO_2_ ink were prepared by dissipating 20 mg Pt/C and 20 mg IrO_2_ powder in the mixture of 950 µL ethanol and 50 µL Nafion with 30 min of ultrasonication. Then the as-prepared ink was coated onto the carbon felt with the loading mass density of about 2.0 mg/cm^2^ and dried at 60 °C. The long-term stability measurements were performed using the chropotentiometric measurements.

### Material characterization

Morphology of the samples was observed by a field-emission SEM (HITACHI S-4700, S-4800) and transmission electron microscopy (TEM, Tecnai G^2^ F30). Raman spectra were measured by a Renishaw RM-1000 Raman microscope using a 514.5 nm laser. X-ray diffraction (XRD, Rigaku D/Max 2500/PC) measurements were carried out to analyze the crystal structure of the samples. X-ray photoelectron spectroscopy (XPS, Escalab 250Xi) is used to probe into the elemental composition and bonding states of the samples. The molar ratio of each component in the composite materials was determined by ICP-MS (Nexlon 300X). The XAS data were acquired at beamline 9-3 (BL9-3) at Stanford Synchrotron Radiation Lightsource (SSRL), SLAC National Accelerator Laboratory. The SPEAR 3 storage ring operated at 500 mA and 3.0 GeV. BL9-3 is equipped with a rhodium-coated vertical collimating mirror upstream of the Si (220) monochromator and an additional downstream rhodium-coated bent cylindrical focusing mirror. Harmonic rejection was accomplished by setting the cut-off angle of the mirrors to an appropriate energy. Incident and transmitted X-rays were monitored using gas ionization chambers and X-ray absorption was measured as the primary fluorescence excitation spectrum using an array of 100-element Ge detector. All absorption spectra, μ(E), were deglitched and corrected for detector dead time using the custom LABVIEW software at BL 9-3. Further data reduction was performed using IFEFFIT-based Athena software programs. For each measurement, three scans were conducted and the average values were used to obtain the final spectrum. All spectra were edge-step normalized using pre and post-edge backgrounds. The edge energy, *E*_0_, was set to 1.0 and determined by the energy of the highest peak in the first derivative of μ(*E*).

### Calculating method

The present first principle DFT calculations were performed by Vienna Ab initio Simulation Package (VASP)^60^ with the projector augmented wave (PAW) method^61^. The exchange-functional is treated using the generalized gradient approximation (GGA) of Perdew–Burke–Ernzerhof (PBE) functional^60^. The cut-off energy of the plane-wave basis is set at 500 eV for calculations of atoms and cell optimization. The vacuum spacing in a direction perpendicular to the plane of the catalyst is at least 15 Å. The Brillouin zone integration is performed using 3 × 3 × 1 Monkhorst-Pack k-point sampling for a primitive cell^62^. A convergence energy threshold of 10^−5^ eV was used during the self-consistent calculations. The equilibrium lattice constants were optimized with maximum stress on each atom within 0.05 eV/Å. The Hubbard *U* (DFT + *U*) corrections for 3d transition metal were made according to the literature^63–65^. According to the previous works with similar structure^63^, based on the traditional DFT method, the U values of 3d-transition metals of Fe, Co, and Ni were chosen as 3.0, 3.5, and 3.0, respectively. During the calculation, the free energy changes of the four steps for OER were investigated (*ΔG*_*I*_*, ΔG*_*II*_*, ΔG*_*III*_*, ΔG*_*IV*_). The calculating formula of the free energy is as follows:

6 $${\it{{{\Delta}}}}G = {\it{{{\Delta}}}}E_{{\mathrm{DFT}}} + {\it{{{\Delta}}}}E_{{\mathrm{ZPE}}}\, {\mathrm{ - }}\, T \times {\it{{{\Delta}}}}S$$

Where Δ*G* is the free energy changes of each steps, Δ*E*_DFT_ is the change in total energy obtained from DFT calculations, Δ*E*_ZPE_ is the change in zero-point energy, ΔS is the change in entropy, *T* is temperature. The values used for corrections of Δ*E*_ZPE_ and Δ*S* were quoted from previous work^64^ and listed in Supplementary Table 6.

## Supplementary information

Supplementary Information

## Data Availability

The data that support the plots within this paper and other findings of this study are available from the corresponding author upon reasonable request.
