# Supplementary material for: Graphene/MoS2/FeCoNi(OH)x and Graphene/MoS2/FeCoNiPx multilayer-stacked vertical nanosheets on carbon fibers for highly efficient overall water splitting
Source: Nat Commun. 2021 Mar 2;12:1380. doi: 10.1038/s41467-021-21742-y (PMC7925597; doi:10.1038/s41467-021-21742-y)
Supplement: Supplementary file 1 — Supplementary Information [file 41467_2021_21742_MOESM1_ESM.pdf]

Supplementary Information

Graphene/MoS_2/FeCoNi$(OH)_x$ and Graphene/MoS_2/FeCoNiP_x multilayer-stacked vertical nanosheets on carbon fibers for highly efficient overall water splitting

Xixi Ji, Yanhong Lin, Jie Zeng, Zhonghua Ren, Zijia Lin, Yongbiao Mu, Yejun Qiu, Jie Yu**

Shenzhen Engineering Lab for Supercapacitor Materials, Shenzhen Key Laboratory for Advanced Materials, School of Material Science and Engineering, Harbin Institute of Technology, Shenzhen, University Town, Shenzhen 518055, China
*email: yejunqiu@hit.edu.cn, jyu@hit.edu.cn

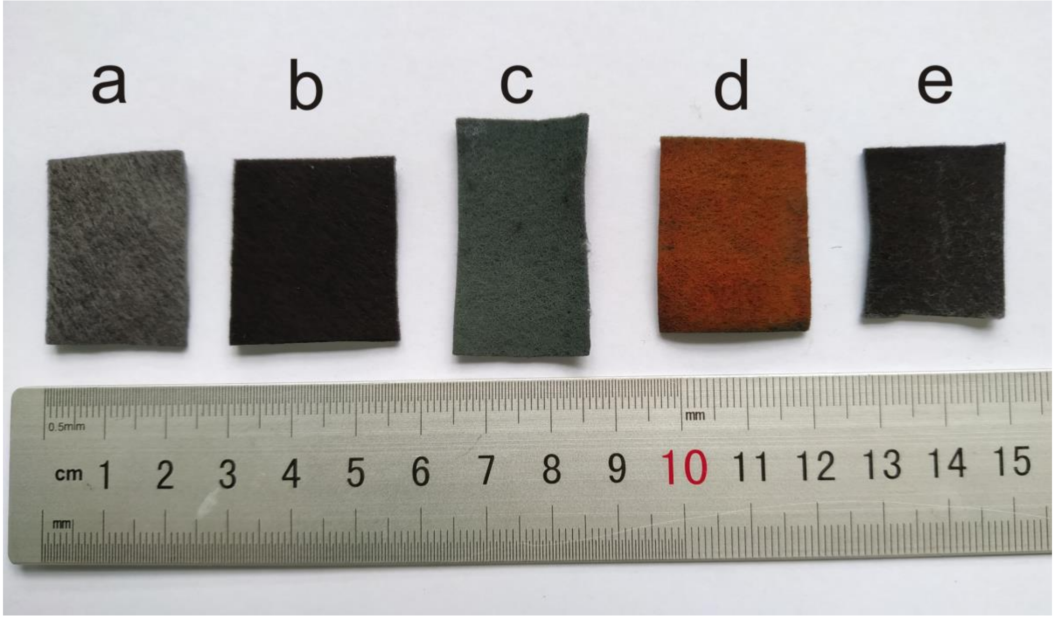

Supplementary Figure 1 | Optical images of different samples. **a** CFs. **b** CF/VGSs. **c** CF/VGSs/MoS_2. **d** CF/VMFO. **e** CF/VMFP.

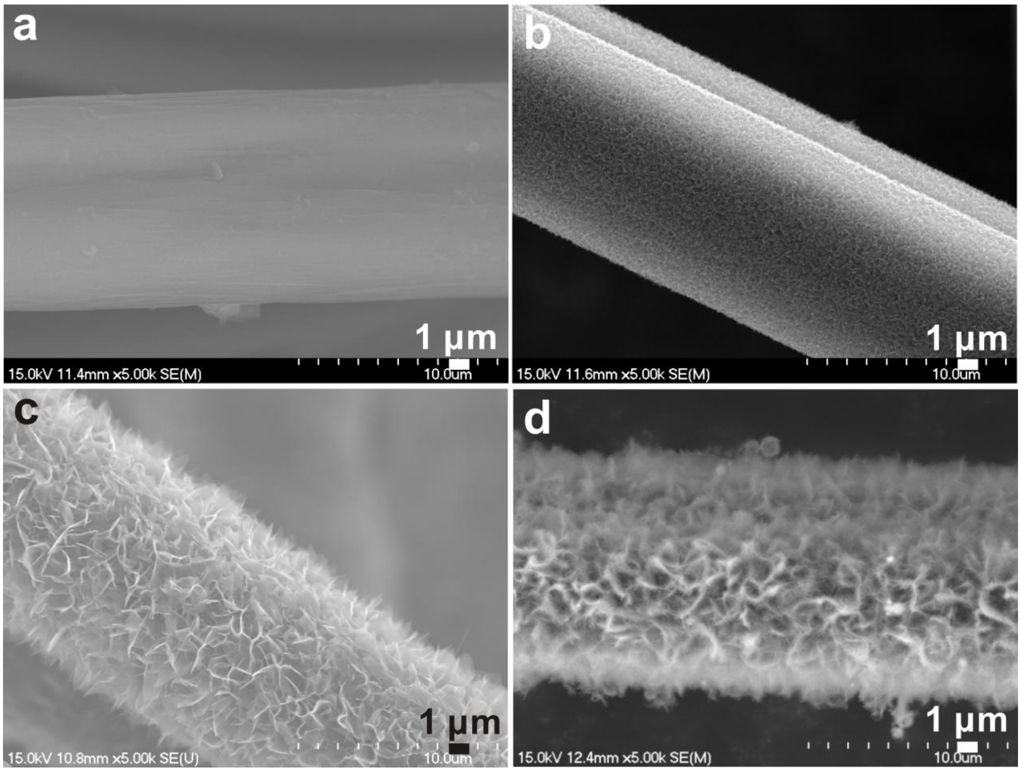

Supplementary Figure 2 | SEM images of different samples. **a** CF. **b** CF/VGSs. **c** CF/VGSs/MoS_2. **d** CF/VMFO.

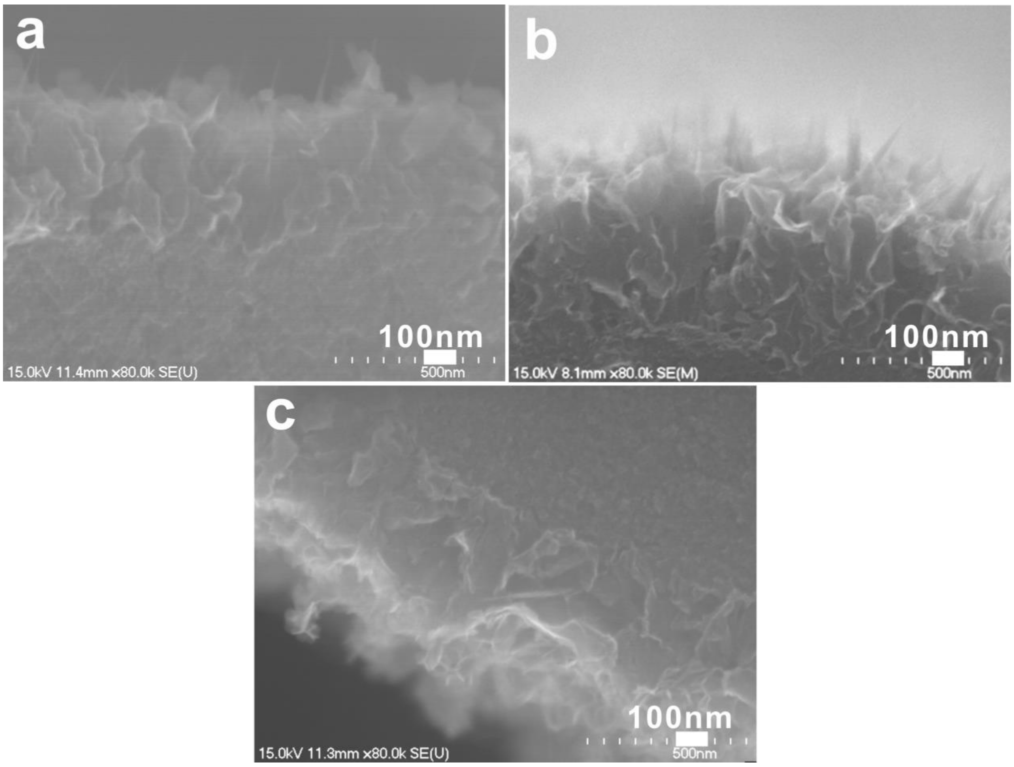

Supplementary Figure 3 | Cross-sectional SEM images of the samples. **a** CF/VGSs. **b** CF/VGSs/MoS_2. **c** CF/VMFO.

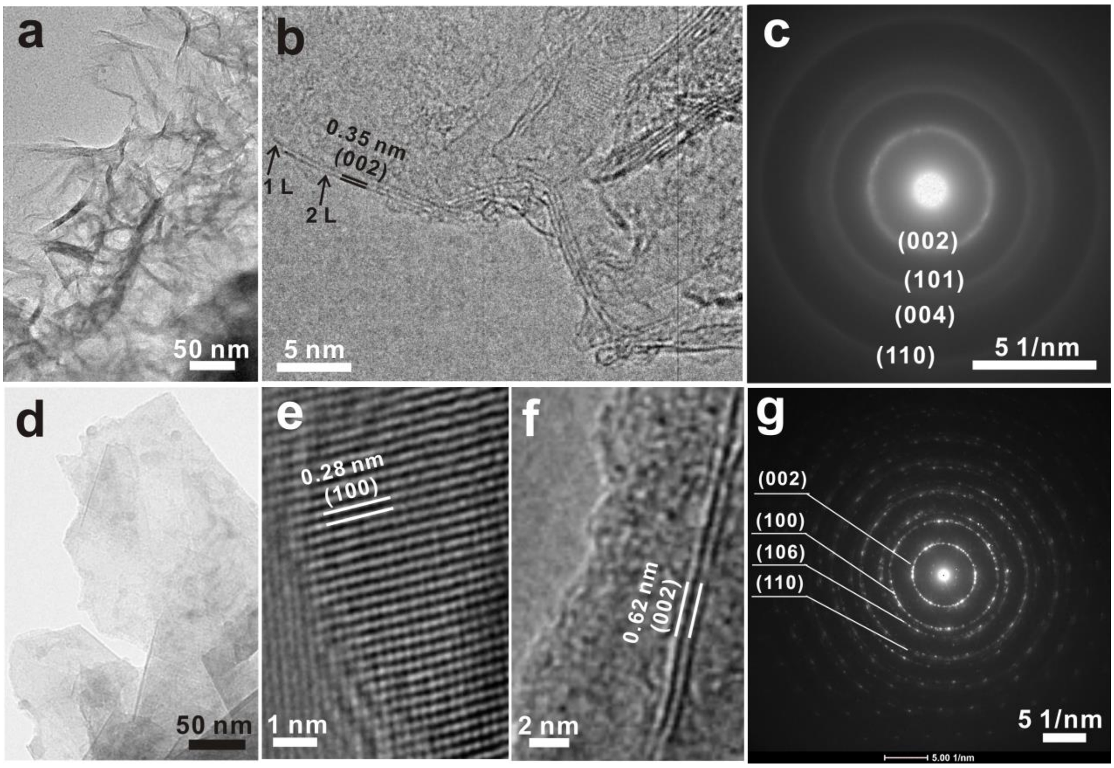

Supplementary Figure 4 | TEM images and SAED patterns of the samples. **a-c** VGSs. **d-g** MoS_2.

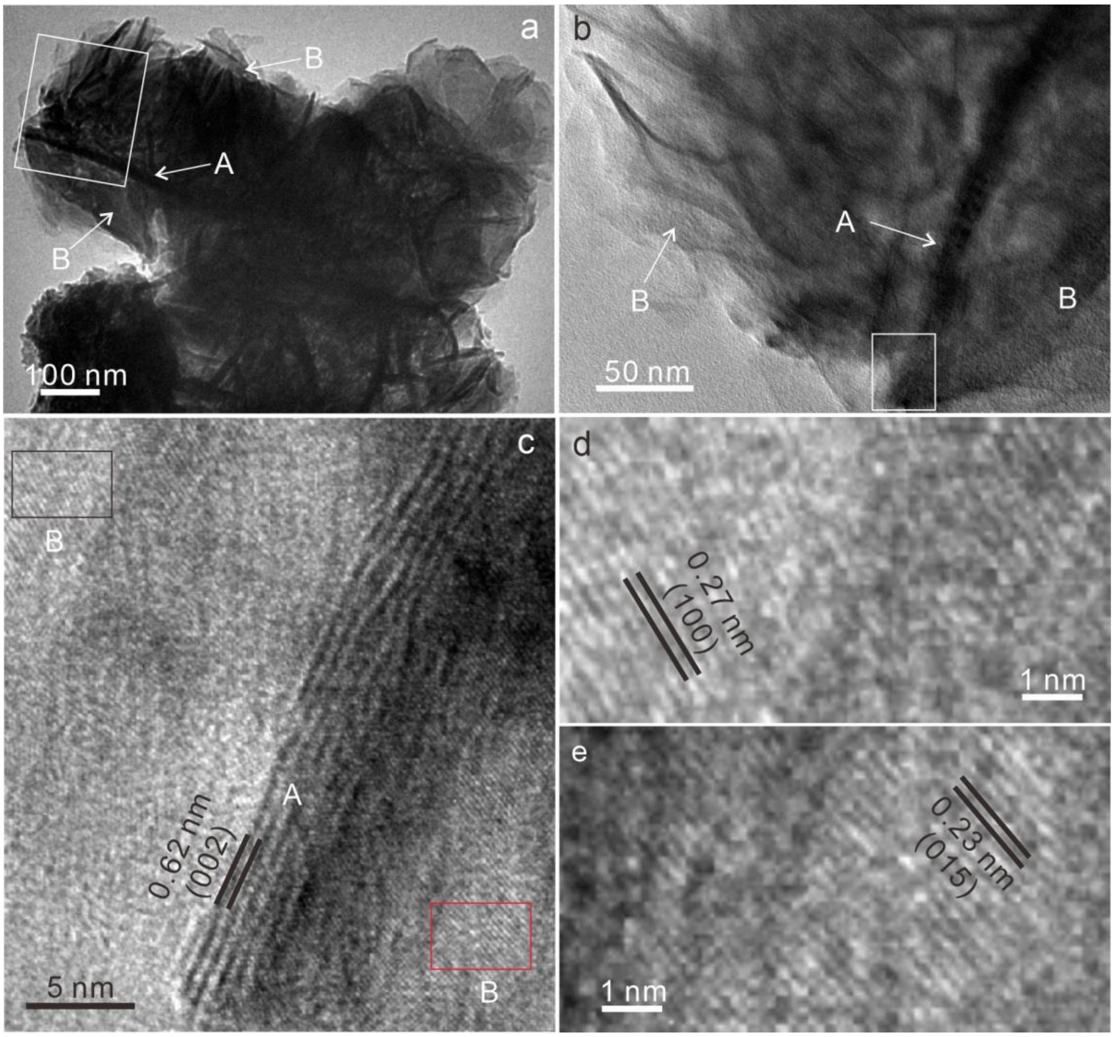

Supplementary Figure 5 | TEM images of $MoS_2/FeCoNi(OH)_x$. (Panel **b** is taken from the squrare area in panel **a**. Panel **c** is taken from the square area in panel **b**. Panel **d** and **e** are taken from the black and red square areas in panel **c**, respectively.)

Supplementary Figure 5 shows TEM images of the $MoS_2/FeCoNi(OH)_x$ nanosheets. In Supplementary Figure 5a and b, it is shown that many nanosheets labeled as B are perpendicular to a nanosheet labeled as A and closely bonded. The HRTEM image (Supplementary Figure 5c) shows that the fringe spacing is 0.62 nm for the A nanosheet, which corresponds to the (002) crystal plane of hexagonal MoS_2. For the B nanosheets, the fringe spacings of 0.27 and 0.23 nm are observed (Supplementary Figure 5d and e), which correspond to (101) and (015) crystal planes of rhombohedral $NiFe(OH)_x$ or hexagonal $NiCo(OH)_x$. This clearly indicates that the $FeCoNi(OH)_x$ nanosheets are grown on the MoS_2 nanosheets, mainly on the side surface.

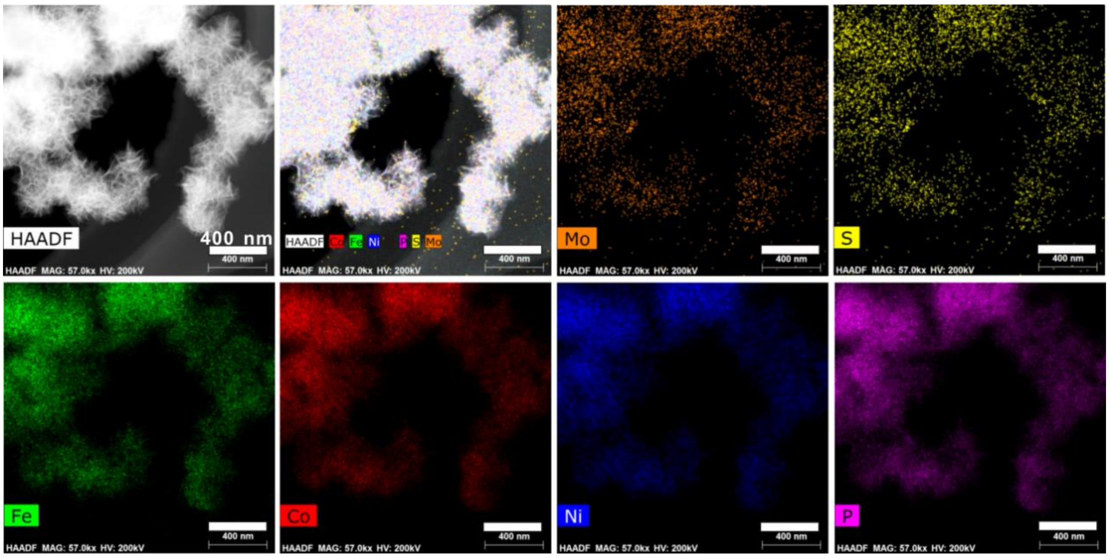

Supplementary Figure 6 | EDS elemental mapping images of MoS_2/FeCoNiPx. Scale bar is 400 nm.

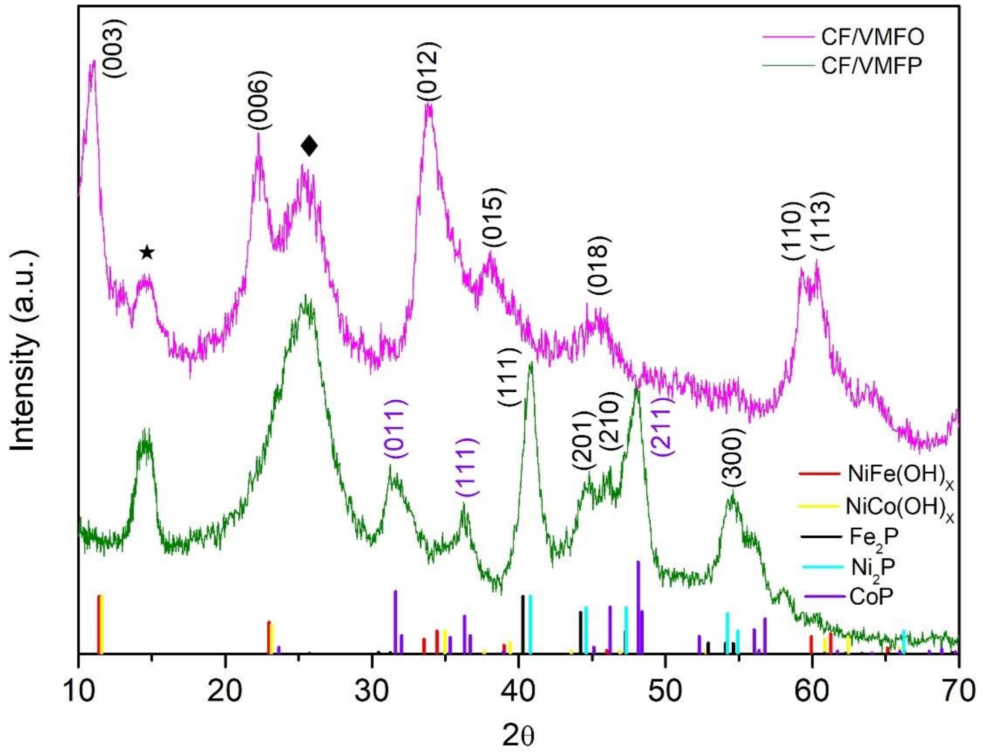

Supplementary Figure 7 | XRD patterns of different samples.

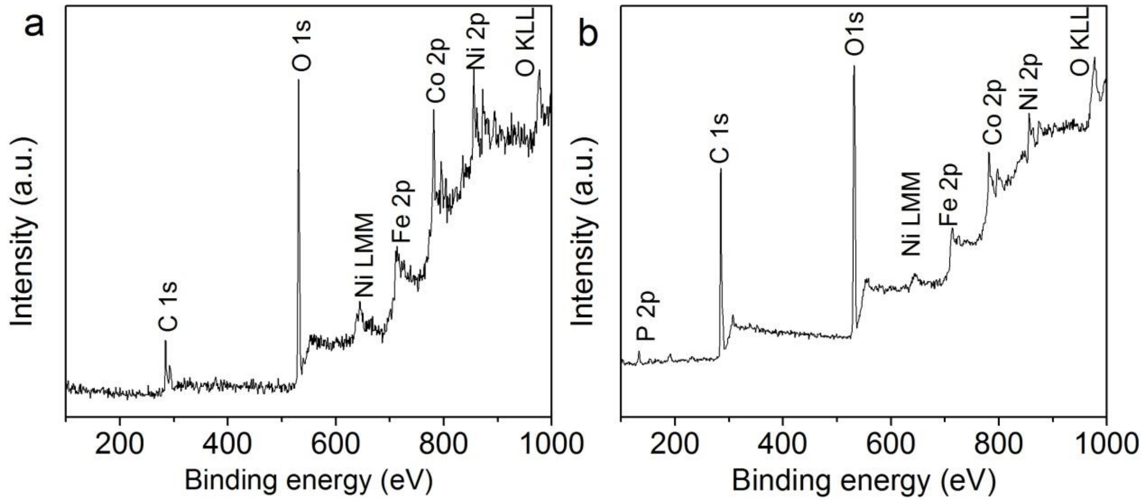

Supplementary Figure 8 | XPS survey spectra of the samples. **a** CF/VMFO. **b** CF/VMFP.

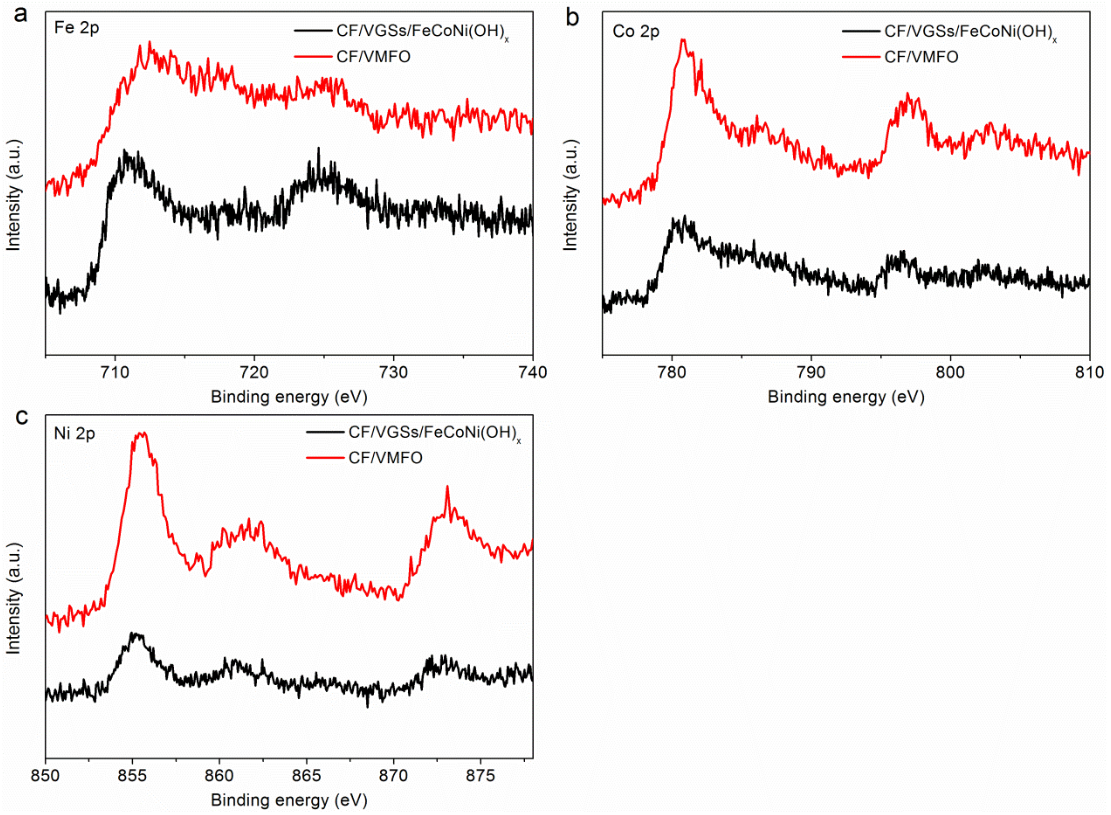

Supplementary Figure 9 | Comparison of XPS spectra for CF/VGSs/FeCoNi(OH)$_x$ and CF/VMFO. **a** Fe 2p. **b** Co 2p. **c** Ni 2p.

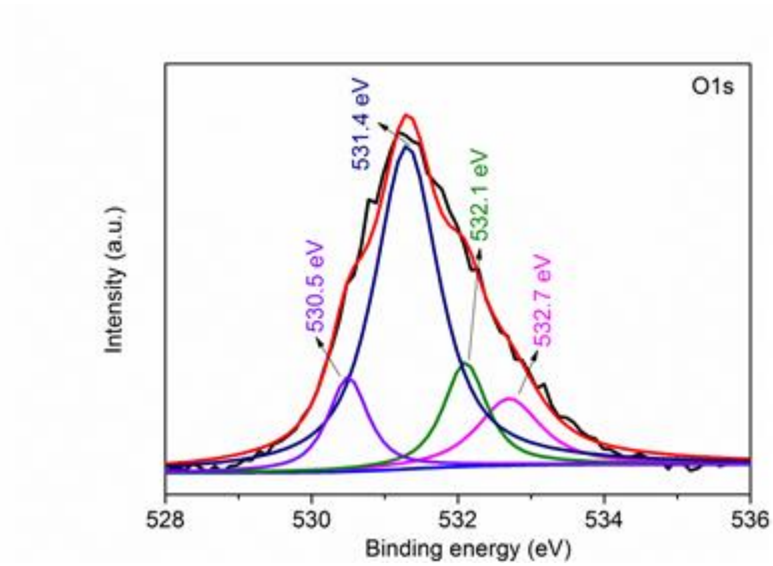

Supplementary Figure 10 | XPS spectra of O 1s for CF/VMFO.

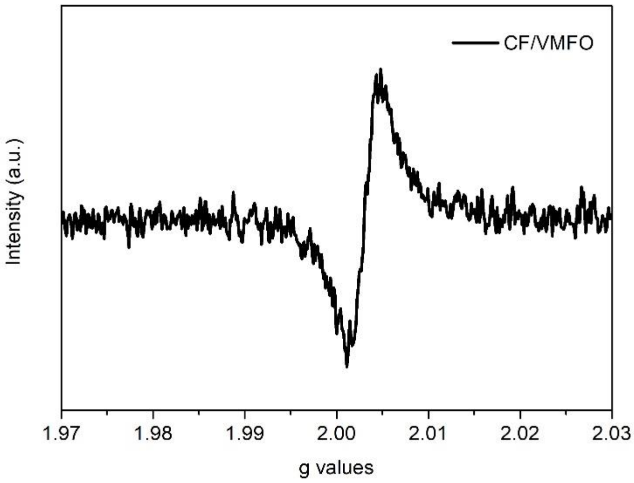

Supplementary Figure 11 | ESR spectra of CF/VMFO.

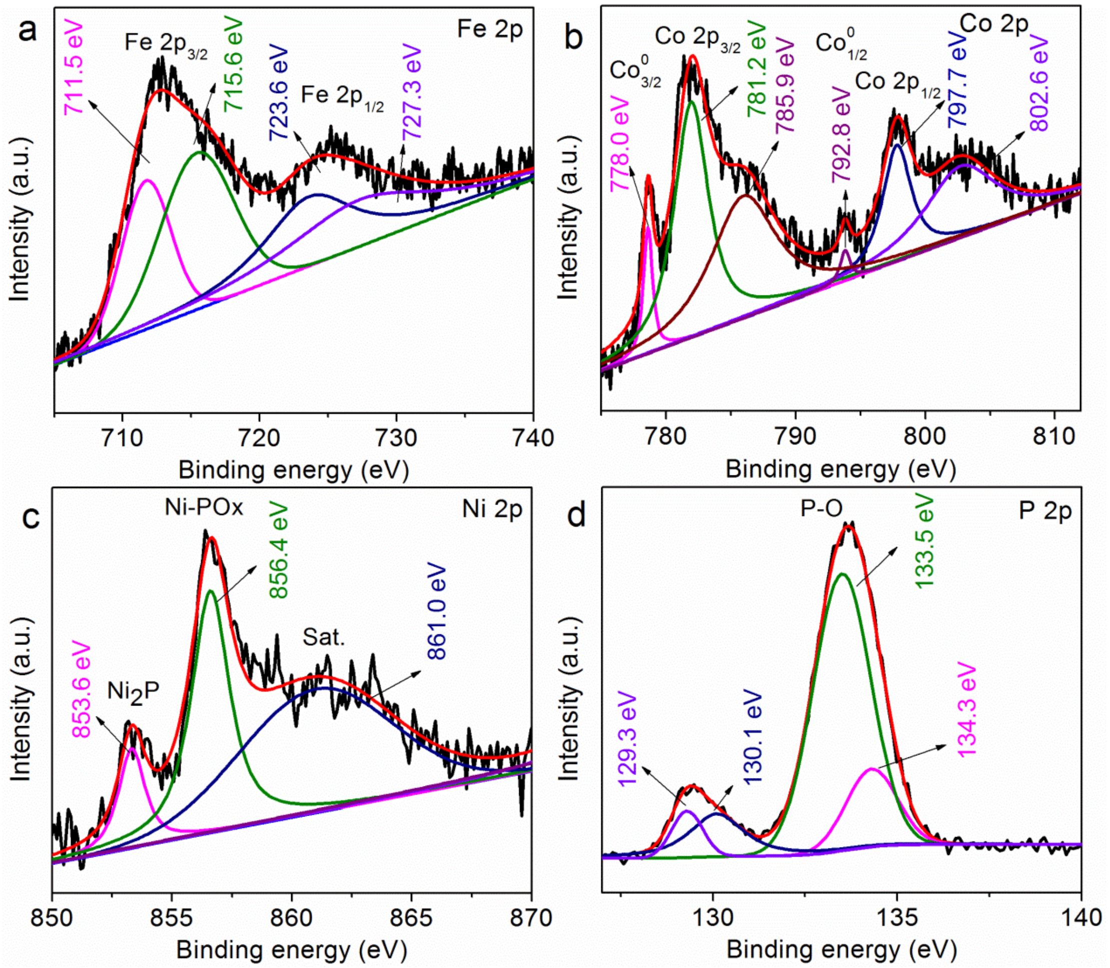

Supplementary Figure 12 | High resolution of XPS spectra for CF/VMFP. **a** Fe 2p. **b** Co 2p. **c** Ni 2p. **d** P 2p.

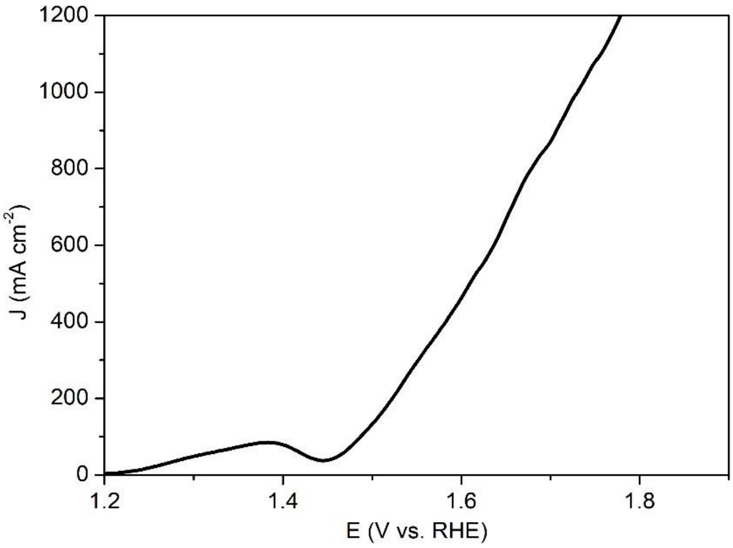

Supplementary Figure 13 | LSV curve of CF/VMFO without IR correction.

Supplementary Table 1 | Comparison of OER performance for the samples of this work and reported catalysts **(**η_{300}-overpotential at 300 mA cm^{-2}, j_{250}-current density at 250 mV overpotential with iR correction, η'_{100}-overpotential at 100 mA cm^{-2} without iR correction, η'_{500}-overpotential at 500 mA cm^{-2} without iR correction).

catalysts	Electrolyte	η_{300} (mV)	Tafel slope (mV dec^{-1})	j_{250} (mA cm^{-2})	η'_{100} (mV)	η'_{500} (mV)	Ref.
CF/VMFO	1 M KOH	215	33.0	1297	257	377	This work
CF/IrO_2	1 M KOH	>450	64.2	7	/	/	This work
Cu@NiFe LDH	1 M KOH	300*	27.8	40*	/	/	1
EG/$Co_{0.85}$Se/NiFe-LDH	1 M KOH	/	57	80*	/	/	2
FeP/Ni_2P	1 M KOH	252*	22.7	250*	/	/	3
NiFe-OH/NiFeP/NF	1 M KOH	258*	39	290*	/	/	4
$Co(OH)_2$@NCNTs@NF	1 M KOH	/	72	/	400*	/	5
$(CoNi)_{0.85}$Se/NiCo LDH	1 M KOH	/	77	20*	/	/	6
Fe-CoP/NF	1 M KOH	270*	36	215*	/	/	7
e-ICLDH@GDY/NF	1 M KOH	260*	43.6	100*	/	/	8
Ni_2P-Ni_3S_2 HNAs/NF	1 M KOH	370*	62	35*	/	/	9
MoS_2/FNS/FeNi foam	1 M KOH	/	28.1	200*	/	/	10
S:CoP@NF	1 M KOH	/	82	8*	/	/	11
NiMoN@NiFeN	1 M KOH	310*	58.6	75*	/	/	12
A-Ir_1/$Co_{0.8}Fe_{0.2}Se_2$	1 M KOH	290*	/	55*			13
$NiMoO_x$/NiMoS	1 M KOH	240*	34	390*	/	/	14
$W_{0.2}Er_{0.1}Ru_{0.7}O_{2-\delta}$	0.5 M H_2SO_4	250*	66.8	300*	/	/	15
S-(Ni,Fe)OOH	1 M KOH	310*	48.9	30	/	/	16
NiFe-MOF array	0.1 M KOH	/	34	/	270*	500*	17
NiFe LDH@NiCoP/NF	1 M KOH	/	46	/	340*	/	18
Fe-Ni/NF	1 M KOH	/	/	/	270*	/	19
Ni_3N-NiMoN-5	1 M KOH	/	64	/	490*	/	20
Ni-P-B/Paper	1 M KOH	335*	/	<50	370*	/	21
Fe-Co-P nanoboxes	1 M KOH	/	31	10*	360*	/	22

*The data were calculated according to the curves given in the literature.

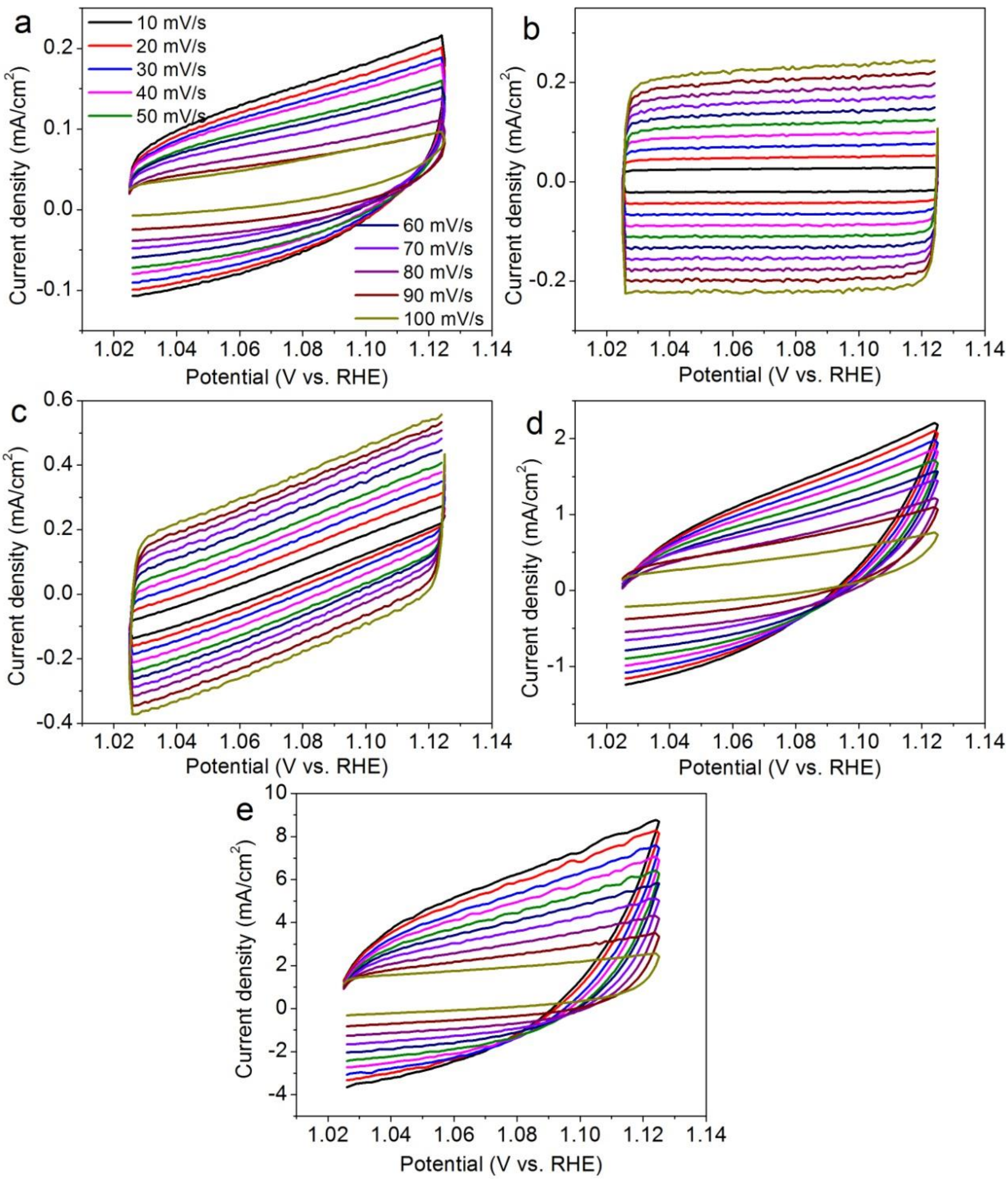

Supplementary Figure 14 | CV curves of different samples. **a** CF. **b** CF/VGSs. **c** CF/VGSs/MoS_2. **d** CF/VGSs/FeCoNi$(OH)_x$. **e** CF/VMFO.

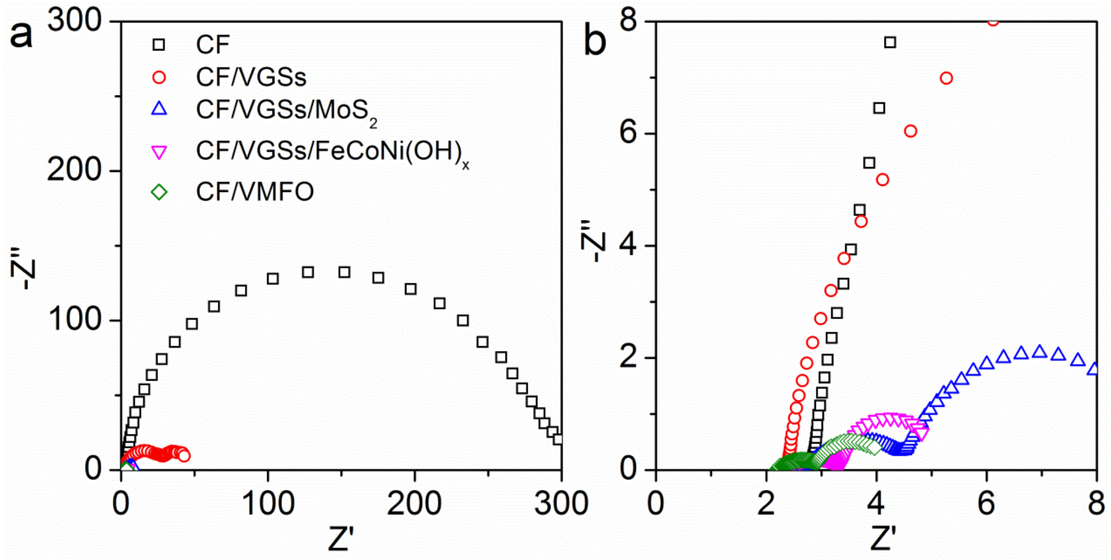

Supplementary Figure 15 | Nyquist plots of different samples. **a** In large axis range. **b** In small axis range (overpotential = 300 mV).

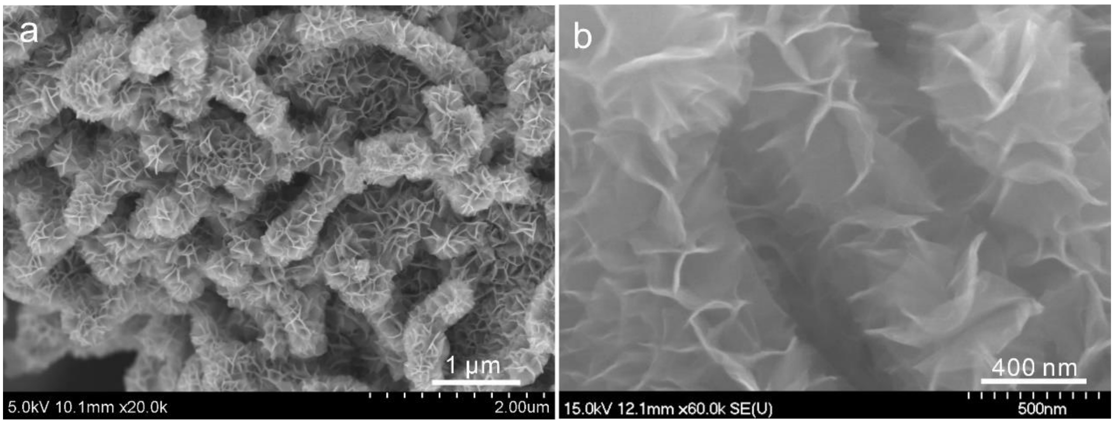

Supplementary Figure 16 | SEM images of CF/VMFO after stability test.

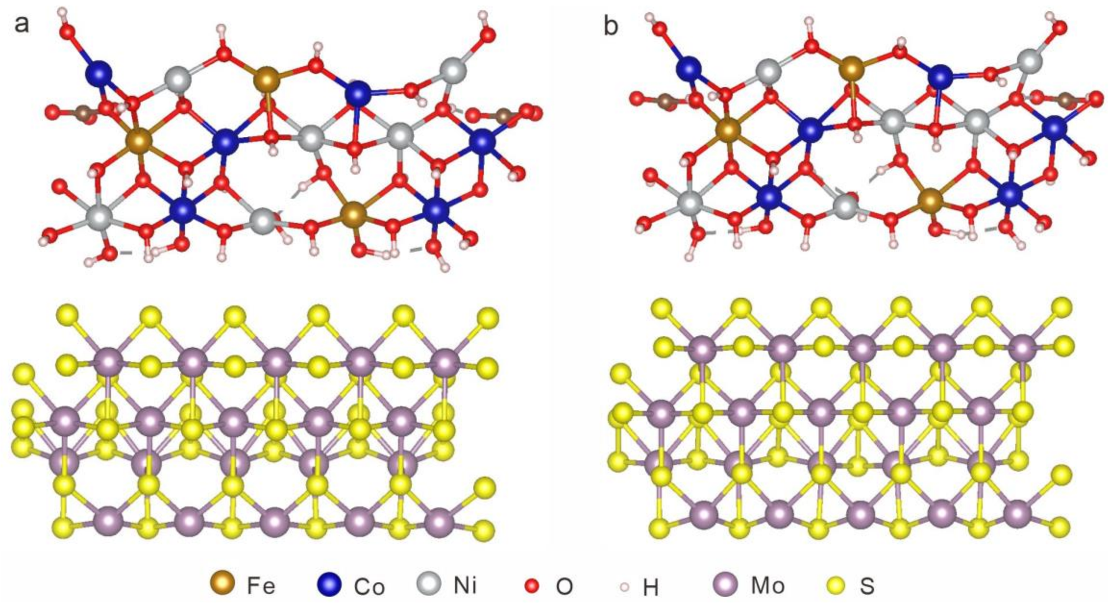

Supplementary Figure 17 | Structural model of MoS_2/FeCoNi$(OH)_x$. **a** Before optimization. **b** After optimization.

Supplementary Table 2 | Gibbs free energy changes of each step and overpotential in the 4e$^-$ mechanism of OER at Fe, Co, and Ni ion positions of (100) planes for FeCoNi$(OH)_x$ and MoS_2/FeCoNi$(OH)_x$.

Samples	ΔG_I	ΔG_{II}	ΔG_{III}	ΔG_{IV}	Overpotential
Fe(100)-FeCoNi$(OH)_x$	0.64	1.64	1.12	1.52	0.41
Fe(100)-MoS_2/FeCoNi$(OH)_x$	0.46	1.30	1.56	1.60	0.37
Co(100)-MoS_2/FeCoNi$(OH)_x$	-0.22	1.22	2.17	1.75	0.94
Ni(100)-MoS_2/FeCoNi$(OH)_x$	-0.39	1.26	2.27	1.78	1.04

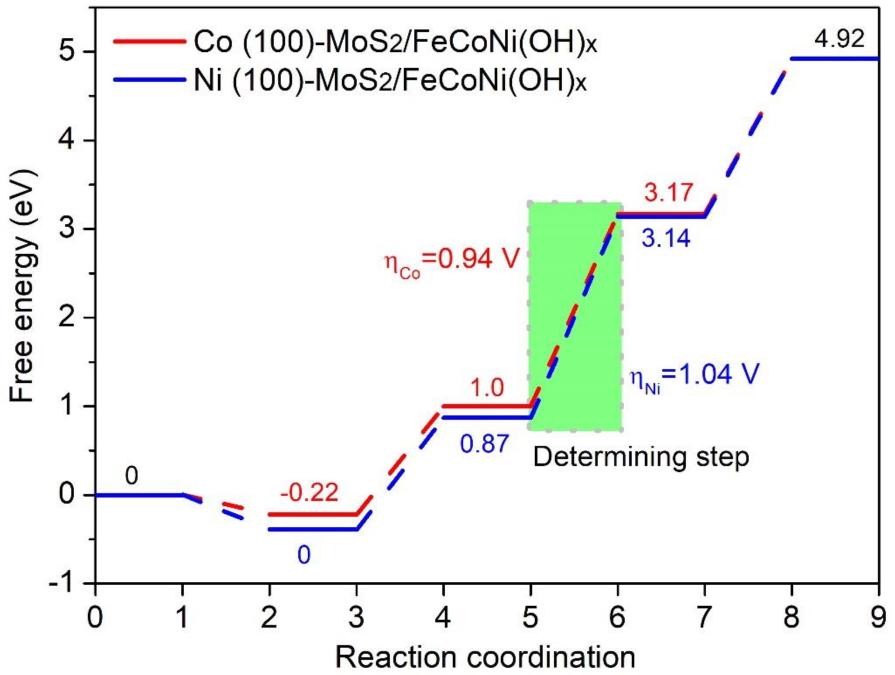

Supplementary Figure 18 | Gibbs free energy diagram for the four steps of OER on $FeCoNi(OH)_x$ and $MoS_2/FeCoNi(OH)_x$ at the positions of Co and Ni ions.

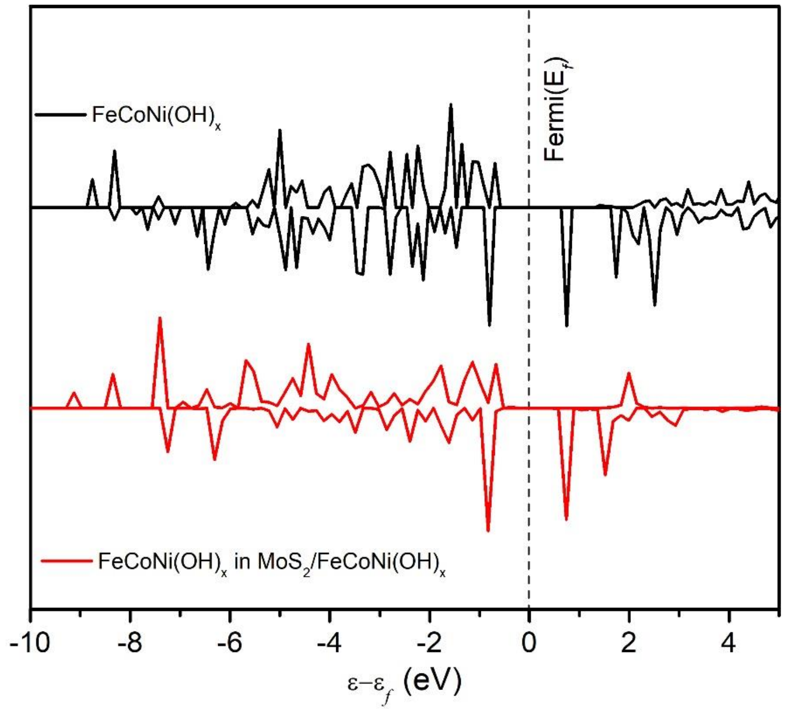

Supplementary Figure 19 | Total density of states curve of $FeCoNi(OH)_x$ and local density of states curve of $FeCoNi(OH)_x$ in $MoS_2/FeCoNi(OH)_x$.

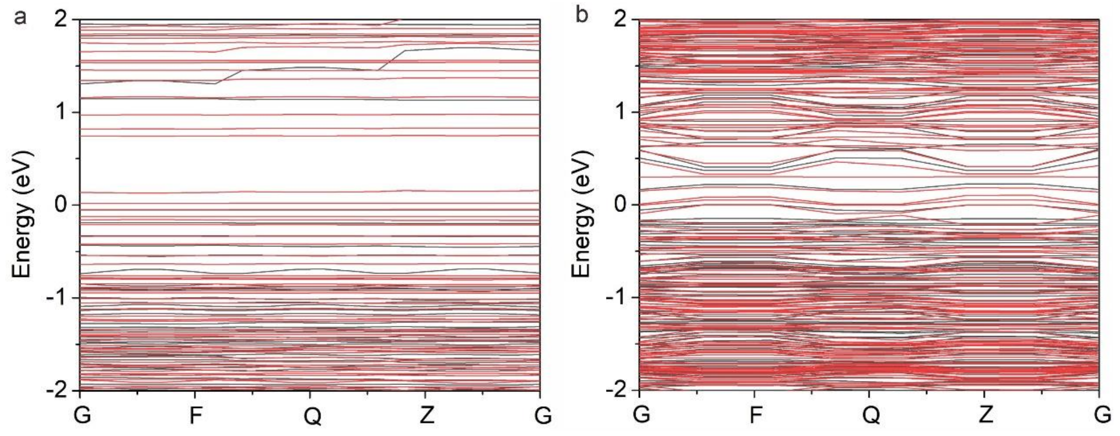

Supplementary Figure 20 | Band structure of different samples. **a** FeCoNi(OH)$_x$, **b** MoS_2/FeCoNi(OH)$_x$.

Supplementary Table 3 | Binding energy of different intermediates in OER on Fe (100) planes.

Samples	*OH	*O	*OOH
Fe(100)-FeCoNi(OH)x	0.64	2.28	3.40
Fe(100)-MoS_2/FeCoNi(OH)x	0.46	1.76	3.32

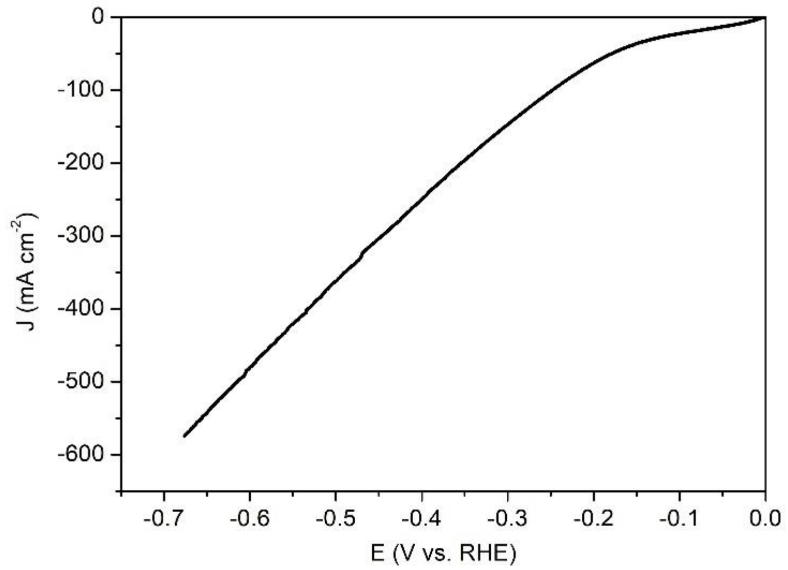

Supplementary Figure 21 | LSV curve of CF/VMFP without IR correction.

Supplementary Table 4 | Comparison of HER performance for the samples of this work and reported catalysts. (**η_{10}**-overpotential at 10 mA cm^{-2}, j_{150}-current density at 150 mV overpotential.)

Catalysts	Electrolyte	η_{10} (mV)	j_{150} (mA cm^{-2})	Tafel slope (mVdec^{-1})	Reference
CF/VMFP	1 M KOH	43	153	25.2	This work
CF/Pt/t	1 M KOH	37	154	73.1	This work
NiFeRu-LDH	1 M KOH	29	/	31	23
Ni_2P-Ni_3S_2 HNAs/NF	1 M KOH	80	58*	65	9
FeP/Ni_2P	1 M KOH	14	140*	24.2	3
MoS_2/Mo_2C	1 M KOH	/	120*	44	24
Co_3S_4@MoS_2	1 M KOH	136	18*	74	25
Holey NiCoP NS	1 M KOH	58	150	57	26
Co/CoP	1 M KOH	253	3*	73.8	27
CoP/CNTs	1 M KOH	76	/	67	28
RuCo alloy	1 M KOH	28	/	31	29
$NiCo_2Px$/CF	1 M KOH	58	240*	34.3	30
Fe-doped CoP	1 M KOH	230	80*	75	31
Ni-Fe/TiN/CC	1 M KOH	75	60*	73	32
S:CoP@NF	1 M KOH	109	33*	54	11
Fe-CoP/NF	1 M KOH	78	48*	92	7
MoS_2/FNS/FeNi foam	1 M KOH	122	15*	45.1	10

*The data were calculated according to the curves given in the literature.

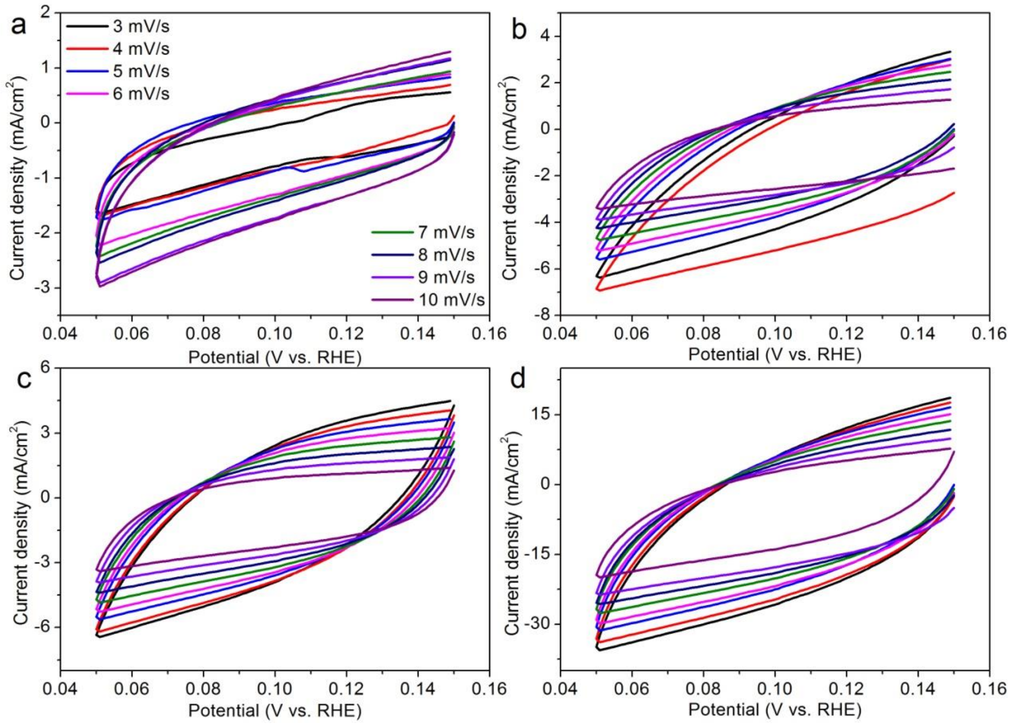

Supplementary Figure 22 | CV curves of different samples. **a** CF/MoS_2. **b** CF/VGSs/MoS_2. **c** CF/VMFO. **d** CF/VMFP.

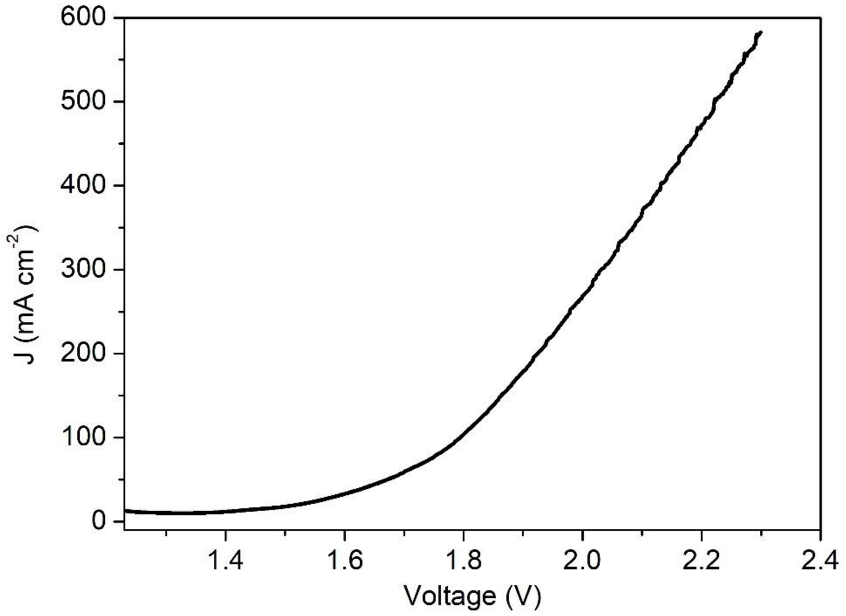

Supplementary Figure 23 | LSV curve of CF/VMFO(+)//CF/VMFP(-) without IR correction.

Supplementary Table 5 | Comparison of water splitting activity of CF/VMFO(+)//CF/VMFP(-) cell in this work with other reported electrocatalysts in 1M alkaline solution (V_{10}-cell voltage at 10 mA cm^{-2}, V_{100}-cell voltage at 100 mA cm^{-2}).

Catalysts	Support	V_{10} (V)	V_{100} (V)	Reference
CF/VMFO (+)//CF/VMFP(-)	Carbon fibers	1.38	1.59	This work
Cu@NiFe LDH(+)//Cu@NiFe LDH(-)	Cu foam	1.54	1.69	[1]
Ni_3FeN/r-GO(+)//Ni_3FeN/r-GO(-)	Ni foam	1.6	1.96*	[33]
Electrodeposited CoP(+)//Electrodeposited CoP(-)	Cu foil	1.64*	1.744	[34]
EG/$Co_{0.85}$/NiFe-LDH(+)//EG/$Co_{0.85}$/NiFe-LDH(-)	Exfoliated graphene	1.67	/	[2]
NiFe LDH@DG10(+)//NiFe LDH@DG10(-)	Ni foam	1.43*	/	[35]
FeP/Ni_2P(+)//FeP/Ni_2P(-)	Ni foam	1.42	1.6	[3]
e-ICLDH@GDY/NF(+)// e-ICLDH@GDY/NF(-)	Ni foam	1.43	1.47	[8]
S:CoP@NF(+)//S:CoP@NF(-)	Ni foam	1.62*	1.78	[11]
Ni_2P-Ni_3S_2 HNAs/NF(+)//Ni_2P-Ni_3S_2 HNAs/NF(-)	Ni foam	1.5	1.62	[9]
Cu@NiFe LDH(+)//$Ni_{2(1-x)}Mo_{2x}$p(-)	Ni foam	1.51	1.65	[30]
NiFeOx(+)//NiFe-P(-)	Fe foam	/	1.76*	[36]
$MoNi_4$ (+)/MoS_2//Ni_3S_2(-)	Ni foam	1.47	1.67	[37]

*The data were calculated according to the curves given in the literature.

Supplementary Table 6 | Values used for the entropy and zero-point energy corrections in determining the free energy of intermediate species and reactant adsorbed on catalysts.

Species	$T\times\Delta S$ *(eV) (298K)*	ΔE_{ZPE} (eV)
O*	0	0.07
OH*	0	0.33
OOH*	0	0.43
H_2O	0.58	0.57

Reference

1. Yu, L. *et al.* Cu nanowires shelled with NiFe layered double hydroxide nanosheets as bifunctional electrocatalysts for overall water splitting. *Energy Environ. Sci.* **10**, 1820-1827 (2017).
2. Zhao, Y. *et al.* N-P-O co-doped high performance 3D graphene prepared through red phosphorous-assisted "cutting-thin" technique: A universal synthesis and multifunctional applications. *Nano Energy* **28**, 346-355 (2016).
3. Yu, F. *et al.* High-performance bifunctional porous non-noble metal phosphide catalyst for overall water splitting. *Nat. Commun.* **9**, 2551 (2018).
4. Liang, H. *et al.* Amorphous NiFe-OH/NiFeP electrocatalyst fabricated at low temperature for water oxidation applications. *ACS Energy Lett.* **2**, 1035-1042 (2017).
5. Guo, P. *et al.* A highly stable bifunctional catalyst based on 3D $Co(OH)_2$@NCNTs@NF towards overall water-splitting. *Nano Energy* **47**, 96-104 (2018).
6. Xia, C., Jiang, Q., Zhao, C., Hedhili, M. N. & Alshareef, H. N. Selenide-based electrocatalysts and scaffolds for water oxidation applications. *Adv. Mater.* **28**, 77-85 (2016).
7. Cao, L. *et al.* Fe-CoP electrocatalyst derived from a bimetallic prussian blue analogue for large-current-density oxygen evolution and overall water splitting. *Adv. Sci.* **5**, 1800949 (2018).
8. Hui, L. *et al.* Overall water splitting by graphdiyne-exfoliated and -sandwiched layered double-hydroxide nanosheet arrays. *Nat. Commun.* **9**, 5309 (2018).
9. Zeng, L. *et al.* Three-dimensional-networked Ni_2P/Ni_3S_2 heteronanoflake arrays for highly enhanced electrochemical overall-water-splitting activity. *Nano Energy* **51**, 26-36 (2018).
10. Wu, Y. *et al.* Coupling interface constructions of $MoS_2/Fe_5Ni_4S_8$ heterostructures for efficient electrochemical water splitting. *Adv. Mater.* **30**, 1803151 (2018).

11. Anjum, M. A. R. *et al.* Bifunctional sulfur-doped cobalt phosphide electrocatalyst outperforms all-noble-metal electrocatalysts in alkaline electrolyzer for overall water splitting. *Nano Energy* **53**, 286-295 (2018).
12. Yu, L., Zhu, Q., Song, S., Mcelhenny, B. & Ren, Z. Non-noble metal-nitride based electrocatalysts for high-performance alkaline seawater electrolysis. *Nat. Commun.* **10**, 5103 (2019).
13. Zhang, Z., Feng, C., Liu, C., Zuo, M. & Zeng, J. Electrochemical deposition as a universal route for fabricating single-atom catalysts. *Nat. Commun.* **11**, 1215 (2020).
14. Zhai, P. *et al.* Engineering active sites on hierarchical transition bimetal oxides/sulfides heterostructure array enabling robust overall water splitting. *Nat. Commun.* **11**, 5462 (2020).
15. Hao, S. *et al.* Dopants fixation of Ruthenium for boosting acidic oxygen evolution stability and activity. *Nat. Commun.* **11**, 5368 (2020).
16. Yu, L. *et al.* Ultrafast room-temperature synthesis of porous S-doped Ni/Fe (oxy)hydroxide electrodes for oxygen evolution catalysis in seawater splitting. *Energy Environ. Sci.* **13**, 3439-3446 (2020).
17. Duan, J., Chen, S. & Zhao, C. Ultrathin metal-organic framework array for efficient electrocatalytic water splitting. *Nat. Commun.* **8**, 15341 (2017).
18. Zhang, H. *et al.* Bifunctional heterostructure assembly of NiFe LDH nanosheets on NiCoP nanowires for highly efficient and stable overall water splitting. *Adv. Funct. Mater.* **28**, 1706847 (2018).
19. Zhang, G., Wang, G., Liu, H., Qu, J. & Li, J. Rapidly catalysis of oxygen evolution through sequential engineering of vertically layered FeNi structure. *Nano Energy* **43**, 359-367 (2017).
20. Aiping. *et al.* Integrating the active OER and HER components as the heterostructures for the efficient overall water splitting. *Nano Energy* **44**, 353-363 (2018).
21. Hao, W. *et al.* Fabrication of practical catalytic electrodes using insulating and eco-friendly substrates for overall water splitting. *Energy Environ. Sci.* **13,** 102-

110 (2020).

22. Zhang, H., Zhou, W., Dong, J., Lu, X. & Lou, X. Intramolecular electronic coupling in porous iron cobalt (oxy)phosphide nanoboxes enhances the electrocatalytic activity for oxygen evolution. *Energy Environ. Sci.* **12**, 3348-3355 (2019).
23. Chen, G. *et al.* Accelerated hydrogen evolution kinetics on NiFe-layered double hydroxide electrocatalysts by tailoring water dissociation active sites. *Adv. Mater.* **30**, 1706279 (2018).
24. Luo, Y. *et al.* Morphology and surface chemistry engineering toward pH-universal catalysts for hydrogen evolution at high current density. *Nat. Commun.* **10**, 269 (2019).
25. Guo, Y. *et al.* Elaborately assembled core-shell structured metal sulfides as a bifunctional catalyst for highly efficient electrochemical overall water splitting. *Nano Energy* **47**, 494-502 (2018).
26. Fang, Z. *et al.* Dual tuning of Ni-Co-A (A = P, Se, O) nanosheets by anion substitution and holey engineering for efficient hydrogen evolution. *J. Am. Chem. Soc.* **140**, 5241-5247 (2018).
27. Xue, Z. *et al.* Janus Co/CoP nanoparticles as efficient mott-schottky electrocatalysts for overall water splitting in wide pH range. *Adv. Energy Mater.* **7**, 1602355 (2017).
28. Zhang, X. *et al.* Iron-doped cobalt monophosphide nanosheet/carbon nanotube hybrids as active and stable electrocatalysts for water splitting. *Adv. Funct. Mater.* **27**, 1606635 (2017).
29. Su, J. *et al.* Ruthenium-cobalt nanoalloys encapsulated in nitrogen-doped graphene as active electrocatalysts for producing hydrogen in alkaline media. *Nat. Commun.* **8**, 14969 (2017).
30. Zhang, R. *et al.* Ternary $NiCo_2P_x$ nanowires as pH-universal electrocatalysts for highly efficient hydrogen evolution reaction. *Adv. Mater.* **29**, 1605502 (2017).
31. Tang, C. *et al.* Fe-doped CoP nanoarray: A monolithic multifunctional catalyst for highly efficient hydrogen generation. *Adv. Mater.* **29**, 1602441 (2017).

32. Peng, X. *et al.* Ni-doped amorphous iron phosphide nanoparticles on TiN nanowire arrays: An advanced alkaline hydrogen evolution electrocatalyst. *Nano Energy* **53**, 66-73 (2018).

33. Gu, Y. *et al.* Electronic Structure Tuning in Ni_3FeN/r-GO Aerogel toward Bifunctional Electrocatalyst for Overall Water Splitting. *ACS Nano* **12**, 245-253 (2018).

34. Jiang, N., You, B., Sheng, M. & Sun, Y. Electrodeposited cobalt-phosphorous-derived films as competent bifunctional catalysts for overall water splitting. *Angew. Chem. Int. Ed.* **54**, 6251-6254 (2015).

35. Jia, Y. *et al.* A heterostructure coupling of exfoliated Ni-Fe hydroxide nanosheet and defective graphene as a bifunctional electrocatalyst for overall water splitting. *Adv. Mater.* **29**, 1700017 (2017).

36. Wang, J., Ji, L., Zuo, S. & Chen, Z. Hierarchically structured 3D integrated electrodes by galvanic replacement reaction for highly efficient water splitting. *Adv. Energy Mater.* **7**, 1700107 (2017).

37. Zhang, J. *et al.* Efficient hydrogen production on $MoNi_4$ electrocatalysts with fast water dissociation kinetics. *Nat. Commun.* **8**, 15437 (2017).
